# Quantification for non-targeted LC/MS screening without standard substances

**DOI:** 10.1038/s41598-020-62573-z

**Published:** 2020-04-02

**Authors:** Jaanus Liigand, Tingting Wang, Joshua Kellogg, Jørn Smedsgaard, Nadja Cech, Anneli Kruve

**Affiliations:** 1 Institute of Chemistry, Faculty of Science and Technology, University of Tartu, Ravila 14A, 50411 Tartu, Estonia; 2 National Food Institute, Research Group for Analytical Food Chemistry, Technical University of Denmark, Kemitorvet Building 202, Kgs, Lyngby, DK-2800 Denmark; 3 Department of Chemistry & Biochemistry, University of North Carolina at Greensboro, Greensboro, North Carolina 27412 United States; 4 Department of Environmental Science and Analytical Chemistry, Stockholm University, Svante Arrhenius väg 16, 106 91 Stockholm, Sweden

**Keywords:** Mass spectrometry, Cheminformatics

## Abstract

Non-targeted and suspect analyses with liquid chromatography/electrospray/high-resolution mass spectrometry (LC/ESI/HRMS) are gaining importance as they enable identification of hundreds or even thousands of compounds in a single sample. Here, we present an approach to address the challenge to quantify compounds identified from LC/HRMS data without authentic standards. The approach uses random forest regression to predict the response of the compounds in ESI/HRMS with a mean error of 2.2 and 2.0 times for ESI positive and negative mode, respectively. We observe that the predicted responses can be transferred between different instruments via a regression approach. Furthermore, we applied the predicted responses to estimate the concentration of the compounds without the standard substances. The approach was validated by quantifying pesticides and mycotoxins in six different cereal samples. For applicability, the accuracy of the concentration prediction needs to be compatible with the effect (e.g. toxicology) predictions. We achieved the average quantification error of 5.4 times, which is well compatible with the accuracy of the toxicology predictions.

## Introduction

Liquid chromatography mass spectrometry (LC/MS) has become the most versatile analytical tool to discover and detect metabolites^1^, pharmaceuticals and their transformation products^2^, environmental contaminants^3^, and food contaminants^4^ with non-targeted^5^ analysis. To better understand the chemical and mechanistic dynamics of a system, a quantitative approach is preferred, which requires two main elements: identification and quantification of each metabolite (determining the concentration of compounds within the dataset). Currently, accurate mass measurements from high-resolution mass spectrometry (HRMS), together with relevant data analysis^6,7^, are increasingly able to assign putative structures to the detected features^8^. Quantifying, however, remains a primary challenge. For example, out of 114 100 compounds in the Human Metabolome Database, only ca. 21 000 have been detected and identified^9^. Currently, the ability to obtain quantitative information from LC/MS is almost exclusively limited by the availability of standard substances, as different compounds ionize to different extents in an electrospray (ESI) source. The response of the compounds in LC/MS is influenced by the properties of the compound, eluent composition, and instrument. Thus, quantifying all detected compounds with a targeted analysis is exceedingly difficult, as standard reference materials (to match retention time, mass fragmentation pattern, and provide a calibration curve) are not available for the majority of compounds.

Additionally, the results of most LC/MS analyses conducted in different laboratories can currently only be compared based on qualitative data, as the measurement conditions and instruments used vary strongly and quantitative data are not available^5^. The lack of facile quantification also represents an obstacle to longitudinal studies, as samples collected over a long period of time must be stored and analysed all together in the same laboratory with the same methods. This raises concerns about sample preservation and stability, and delays in information dissemination, especially in cases where fast interventions may be crucial.

Several groups have studied ionization efficiencies in electrospray sources to understand which factors contribute to the ionization process^10–15^. Several ionization efficiency prediction models have been proposed for small sets of compounds, mainly based on multilinear regression algorithms (see Table S1 in Supporting Information for a detailed overview). In general, researchers have focussed on finding a suitable predictive model for a specific compound group (drugs, metabolites, steroids) in studies that normally cover a few tens of compounds measured on a single instrument, and most have been carried out in ESI positive mode with one eluent composition in infusion or flow injection mode. More recently, some groups have used LC separation with gradient elution^16^. The latter point is important to effectively model the influence of both compound properties and eluent properties. However, a general model that would fit a wide range of compounds, LC conditions, instruments, and would also allow quantitation is lacking.

Based on the promising results obtained for predicting ionization efficiencies for specific compound classes, we propose a general approach for quantifying compounds using their predicted ionization efficiency values. We demonstrate that this approach can be transferred between different eluents, LC setups, and instruments and can be used for the analysis of real samples. The approach incorporates both positive and negative ionization modes under more than 100 eluent compositions and covers over 450 compounds. We validated this approach by predicting the concentrations of 35 compounds, including pesticides and mycotoxins, in cereal samples. The quantification for the samples was conducted on an instrument that was not involved in the ionization efficiency model development. The results presented herein demonstrate that our approach for calculating ionization efficiencies has the potential for making non-targeted analysis (semi-)quantitative.

## Experimental

### Ionization efficiency data: training and test set

Previously measured ionization efficiency values were collected in positive^17–24^ and negative^24–28^ mode. The ionization efficiency values for both modes and all studied eluent compositions are presented in Table S2. We measured an additional 165 compounds with diverse chemical properties in both the main eluent composition (acetonitrile/0.1% formic acid (aq) 80/20) and ca. 1000 new analyte-eluent combinations. For ESI positive mode, a total of 3139 ionization efficiency values were measured and collected from our previous works^17–21^. These data belong to 353 unique compounds and 106 different eluent compositions (Table S3 in SI). For ESI negative mode, an additional 1286 ionization efficiency values were collected from our previous works^25,26^, including 33 eluent compositions Table S4), and 101 unique compounds.

The compounds covered by the model include protonated and intrinsically charged compounds in positive mode and deprotonated compounds in the negative mode. In ESI positive mode both nitrogen and a significant amount of oxygen bases could be measured. In ESI negative mode compounds with significant acidic moieties could be detected as [M-H]^−^, including amino acids, benzoic acids and derivatives, phenols, amines, heterocycles, guanidines, and diazines (see Table S5 and S6). As we focus on [M]^+^, [M + H]^+^ and [M-H]^−^, some of the compound groups are better represented then some of the other groups. For example, the lipids and sugars form primarily sodiated ions in ESI positive mode and are, therefore, not included in the scope of this approach.

From an application perspective, the training and test set compounds fall into a diverse array of categories, including drugs or drug-like compounds (e.g. terfenadine, ketoconazole, lidocaine), metabolites (e.g. acetylcholine, dopamine, thiamine), amino acids, small organic precursors (amines primarily), lipids (e.g. myristic acid, progesterone, glyceryl tributyrate) as well as industrial dyes (e.g. sudan II, sudan IV). The chemical space covered by these compounds was compared to the chemical databases Drugbank, NORMAN, and the Human Metabolome Database (HMDB), and is presented at Figs. S1 and S2.

### Validation set

The proposed method was validated on a set of 35 pesticides and mycotoxins (Table S7), 28 of which had not been included in the training or test set. The chemical space covered by the validation compounds can be seen from Fig. S3. The compounds were measured at 10 concentration levels in solvent (acetonitrile), oat, barley, rye, wheat, rice, and maize with Agilent 6495 triple quadrupole instrument. The concentrations of the compounds ranged over 5 orders of magnitude from 3.6 nM to 0.35 mM. Altogether, 2233 data points (pesticide, matrix, and concentration combinations) were measured and corresponding concentrations were predicted. The validation set was used to evaluate the applicability of the ionization efficiency predictions for compounds not included in the training or test set. All measurements were also done under gradient separation and on an instrument that was not the primary instrument for ionization efficiency measurements.

### Ionization efficiency measurements

For the evaluation of log*IE* values, the responses of [M + H]^+^ and [M-H]^−^ were recorded in an MS full scan mode corresponding to the polarity mode. In case in-source fragmentation of the compounds occurred, the intensities of the fragment ions peaks were summed with the intensity of the molecular ion peak. Six dilutions of the stock solutions were made (1-, 1.25-, 1.67-, 2-, 2.5-, and 5 times) with the corresponding eluent by the autosampler and delivered to MS in flow injection mode. The injection volume was 10 μL, and the eluent flow rate was 0.2 mL/min. Concentrations in the injected solutions ranged from 10^−4^ M to 10^−9^ M and measurements were conducted in the linear range. Most of the measurements of log*IE* values were carried out using an Agilent XCT ion trap mass spectrometer. Additionally, six mass spectrometers from five vendors were used in three labs around the world (Tartu/Estonia, Lyon/France, and Beerse/Belgium, see Table S8). On all instruments, the default MS and ESI parameters were used. In the case of the ion trap instrument, only the optimized Target Mass (TM) parameter was used^29^.

The absolute ionization efficiency values vary significantly depending on the ionization source geometry, ion optics, day, cleanliness of the ionization source, etc. Therefore, we measured the relative ionization efficiency (*RIE*) of a compound M_1_ relative to anchor compound (M_2_) according to the following equation^26^:

1 $$RIE({M}_{1}/{M}_{2})=\frac{slope({M}_{1})\cdot IC({M}_{1})}{slope({M}_{2})\cdot IC({M}_{2})}$$

where the slope of the signal versus concentration is estimated via linear regression in the linear range of the signal-concentration plot and the *IC* is the sum of relative abundances of isotopologues where highest abundance is taken equal to 100. All measurements were made relative to tetraethylammonium (positive mode) and benzoic acid (negative mode). To make the data easier to present and analyze, the logarithmic scale (log*IE*) was used. The scale in negative mode was anchored to the log*IE* of benzoic acid, taken as 0 in the 0.1% ammonia/acetonitrile 20/80 mixture and the scale in positive mode was anchored to log*IE* of tetraethylammonium, taken as 3.95 in the 0.1% formic acid/acetonitrile 20/80. The log*IE* = 0 corresponds to methyl benzoate in the 0.1% formic acid/acetonitrile 20/80. The log*IE* values for each compound were obtained based on the *RIE* and log*IE* value of the anchor compound.

2 $$\log \,I{E}_{{{\rm{M}}}_{1}}=\,\log \,RIE({M}_{1}/{M}_{2})+\,\log \,I{E}_{{\rm{a}}{\rm{n}}{\rm{c}}{\rm{h}}{\rm{o}}{\rm{r}}}$$

To minimize the influence of possible differences in conditions when measuring M_1_ and anchor compound, two steps were taken: (1) each compound was measured on at least three different runs (on three different days) and the results were averaged, and (2) anchor compound was measured in the beginning and the end of each run on each day. To anchor the scales of other eluent compositions, the MS signal intensities of anchor compound in all eluent compositions were measured in the same day and the log*IE* value of anchor compound in an eluent composition b was calculated using Eq:

3 $$\log \,I{E}_{{\rm{E}}{\rm{b}}}=\,\log (I{E}_{{\rm{E}}{\rm{a}}}\cdot \frac{Signa{l}_{{\rm{E}}{\rm{b}}}}{{c}_{{\rm{E}}{\rm{b}}}}\cdot \frac{{c}_{{\rm{E}}{\rm{a}}}}{Signa{l}_{{\rm{E}}{\rm{a}}}})$$

where the *Signal*_Eb_ and *Signal*_Ea_ are the signal intensities in eluent composition b and a and *c*_Ea_ and *c*_Eb_ are the corresponding concentrations of benzoic acid in the respective eluent compositions.

To develop the model, log*IE* values measured on different instruments were transformed to unified log*IE* values. The unification was performed using the intersection of compounds measured on two instruments in the eluent composition acetonitrile/0.1% formic acid (aq) 80/20^22,23^. To unify the data, linear regression between log*IE* values measured on two instruments was performed (see SI).

### Eluent effect of ionization efficiency

PCA analysis of the physicochemical properties (Fig. S4) of the training and test set compounds (n = 353) was conducted in order to incorporate different eluent compositions to the ionization efficiency predictive model without measuring all of the compounds in all eluents. Next, random sets of 40 compounds were sampled, and the set representing the widest chemical space was chosen for studying the solvent effects. As a result, 20 additional eluent compositions were studied (Table S3), yielding 106 different solvent compositions together with the previously measured eluent conditions. Both acetonitrile and methanol were studied as an organic modifier and the organic modifier percentages studied were 0%, 20%, 50%, 80% and 100%. Water phase additives included formic acid, trifluoroacetic acid, ammonia, ammonium acetate, ammonium formate, ammonium bicarbonate, and ammonium fluoride (pH = 2.0–10.3).

### Model development

#### Data preprocessing

For model development, PaDEL descriptors^30^ (1444 descriptors) were calculated using ChemDes calculator^31^ for every compound. As PaDEL descriptor calculation of some descriptors fails for some compounds, the descriptors with NA (not available values) were removed from the dataset. Next, all the descriptors with the same value for >95% of compounds were eliminated from the dataset. As the third cleaning step, the pairwise correlation of descriptors was considered. If the *R*^2^ was higher than 0.8 the former descriptor was removed from the dataset. After data pre-processing for ESI positive mode 1086 descriptors were left in the dataset and for ESI negative mode 822 descriptors were left in the dataset. Additionally, five empirical eluent descriptors (viscosity^32^, surface tension^33^, polarity index^34^, pH, NH_4_ content (yes/no)) were added to the dataset.

#### Algorithm selection and model development

Different machine learning algorithms (multilinear regression, Ridge regression, support vector machine regression, artificial neural networks and random forest regression) were tested for model development. For each algorithm, a suitable R package (glmnet^35^, e1071^36^, h2o^37^, RRF^38^) was used and the parameters were optimized.

Regularized random forest regression algorithm^38^ from library RRF in R yielded the best prediction accuracy. For ESI negative and positive mode, individual models were developed. For model development, the order of the dataset was randomized and split into two sets. 80% of observations were used for developing the model and 20% of observations were used as a validation set. The number of trees used in the random forest was optimized, with the optimal number was 100 decision trees. The regularization isotherm selected 450 significant descriptors (Table S9) in ESI positive mode and 145 significant descriptors (Table S10) in ESI negative mode.

The code used for training the model is available from Code S1.

#### Concentration from predicted ionization efficiency

As the model output is in universal ionization efficiency values and not instrumentation specific, a set of compounds with known concentrations is used to transform the universal predicted values to instrumentation specific values.

**Figure 1:**
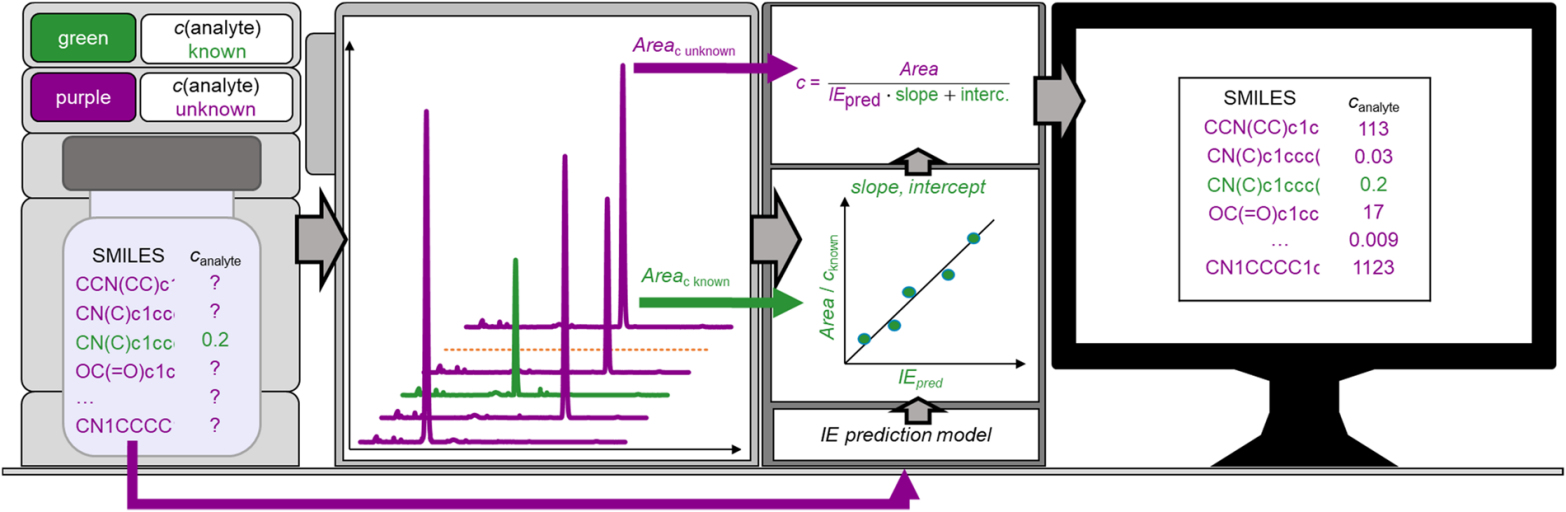
Flow chart of the developed approach to apply ionization efficiency prediction to estimate concentration. Purple is used for compounds of interest and green is used for compounds with known concentration; the latter are used to account for instrument-specific effects in the prediction model.

For transforming the predicted ionization efficiency values a set of compounds with known concentration either spiked to the matrix or as a standard solution was measured in dynamic range with the same method as compounds of interest. Sets of 6, 10, 15, and 31 compounds were tested; see discussion below. From the analysis results logarithmic response factors (*RF*) were calculated:

4 $$\log \,RF=\frac{Signal}{concentration}$$

and correlated with the predicted ionization efficiency values log*IE*_pred_:

5 $$\log \,R{F}_{{\rm{p}}{\rm{r}}{\rm{e}}{\rm{d}}}=slope\cdot \,\log \,I{E}_{{\rm{p}}{\rm{r}}{\rm{e}}{\rm{d}}}+intercept$$

where the signal is the MS1 peak area corrected with isotope distribution. Molar concentrations were used for the calculation of the response factor. Logarithmic response factors were correlated with predicted universal ionization efficiency values to obtain parameters necessary for transforming the predicted ionization efficiency values. The process of concentration prediction is visualized in Fig. 1. For studied pesticides and mycotoxins in cereals, the obtained parameters were used to transform predicted ionization efficiency values to logarithmic response factors. Based on the MS1 peak areas of compounds of interest and predicted response factors it is possible to predict the concentration. Slope and intercept values in Eq. 5 were calculated based on the coefficients of the linear regression curve between log*RF* and log*IE*_pred_ values in the transformation set. In order to validate the obtained results, the prediction errors between real concentration and predicted concentration were calculated.

#### Measuring the prediction accuracy

We express the accuracy of ionization efficiency prediction as a prediction error:

6 $${\rm{prediction}}\,{\rm{error}}=\,\max \{\begin{array}{c}\frac{predicted\,IE}{measured\,IE}\\ \frac{measured\,IE}{predicted\,IE}\end{array}$$

The lowest possible prediction error is 1.0 times and values close to 1.0 times are desirable. In order to illustrate the general prediction accuracy, we use the average error. The accuracy of concentration predictions was estimated with a similar comparison.

#### Sample preparation for cereal samples

All of the cereal samples were prepared according to the QuEChERS extraction method, described elsewhere^39^. In short, the blank samples (pesticides free) were obtained from proficiency test material for the six European Union Proficiency Test EUPTs: EU-PT-CF8 (wheat), C3 (oat), CF10 (rye) and C6 (barley), CF9 (maize), SRM6 (rice). Two grams of homogenized cereal samples were soaked with 10 mL acidified Milli-Q water containing 0.2% formic acid. Then, the sample was extracted with 10 mL of acetonitrile. Thereafter, 4 g of magnesium sulfate and 1 g of sodium chloride were added, and the tube was shaken for 1 min followed by centrifugation. The organic upper layer (2 mL) was removed and shaken with 0.1 g of Bondesil-C18 and 0.3 g of magnesium sulphate for 2 min followed by centrifugation. Then 1.5 mL of purified extract was removed into a vial with insert and spiked with different concentration of tested compounds prior to injection on the LC/MS system.

#### LC/MS analysis of cereal samples

Samples were analyzed on an Agilent 1290 ultrahigh performance liquid chromatograph (Agilent Technologies, CA, U.S.) coupled to an Agilent 6495 triple quadrupole instrument (QQQ) at the University of Tartu (UT). Samples were injected onto an Agilent Zorbax RRHD SB-C18 reversed-phase column (1.8 μm, 2.1 × 50 mm). The mobile phase consisted of (A) water containing 0.1% formic acid and (B) acetonitrile. The analysis was done using a gradient elution at a flow rate of 0.3 mL/min at 30 °C. The gradient was from 5% to 100% B in 7 min, then maintained at 100% for 2 min and returned back to 5% B in 2 min, and maintaining starting conditions at 5% B for 2 min equilibration with 5% B to yield a total runtime of 13 min.

An Agilent’s Jet Stream electrospray ionization (ESI) interface was used in positive ion mode (ESI+) with the following settings: capillary voltage 3 kV, nebulizer pressure 20 psi, sheath gas flow rate 11 L/min, sheath gas temperature 350 °C, dry gas temperature 250 °C, and dry gas flow rate 14 L/min. Spectra were collected from m/z 100 to 1100 Da. The injection volume was 1 μL. Peaks were manually integrated, and peak areas were used for all calculations.

## Results

### Predicting ionization efficiencies

In order to quantify the compounds based on the ionization efficiency values we (1) measured *IE* values for a wide set of compounds, (2) used the measured *IE* values as well as compound and eluent descriptors for developing the model that would allow predicting ionization efficiencies, and (3) validated the approach by using the predicted *IE* to quantify a set of compounds in cereal samples. All of the *IE* values have been measured as relative values; the *IE* of methyl benzoate and benzoic acid have been arbitrarily taken as *IE* = 1 (log*IE* = 0) in ESI positive and negative mode, respectively.

**Figure 2:**
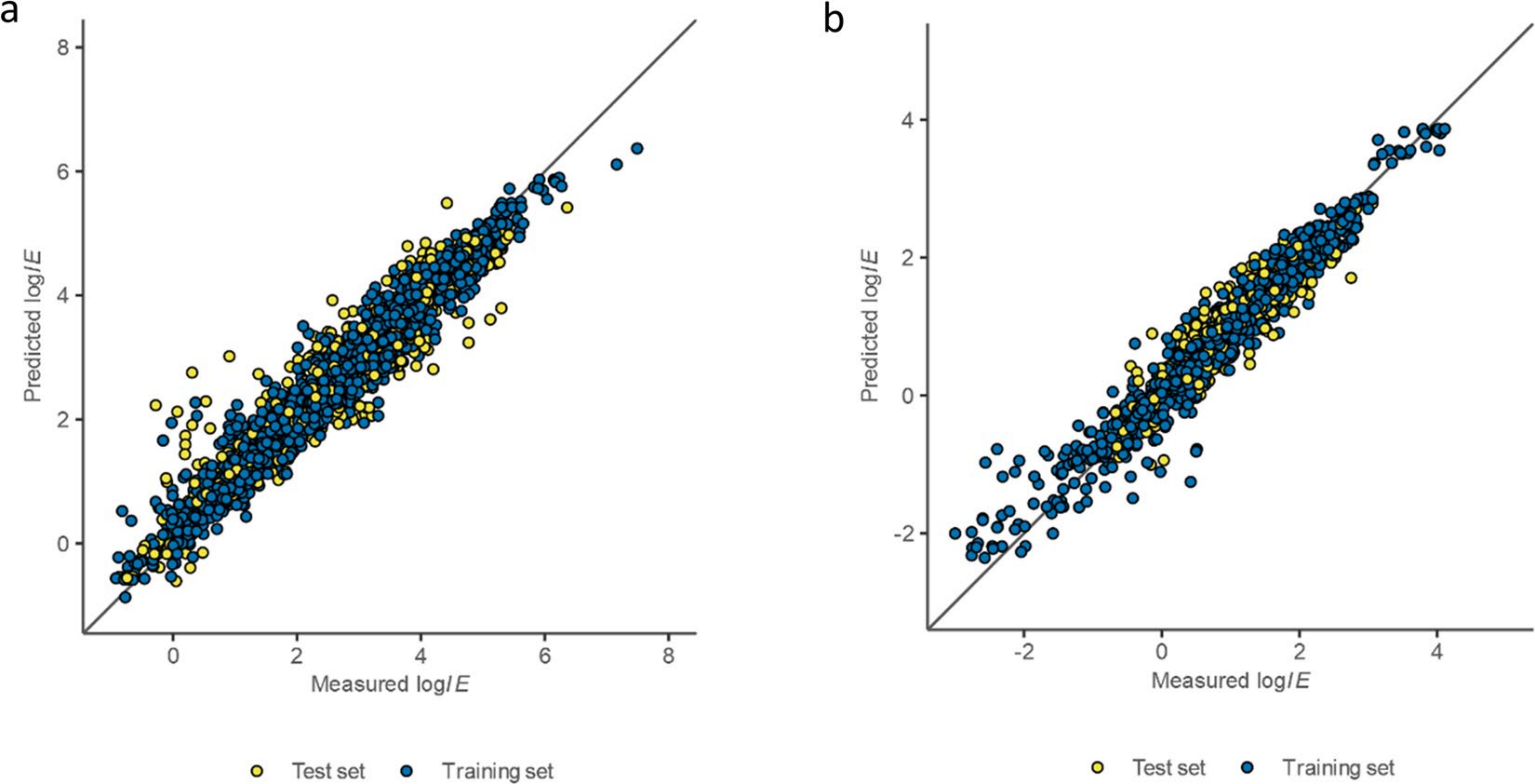
Performance of ionization efficiency prediction models. Black line denotes ideal fit. (**a**) ESI positive mode with 3139 datapoints. (**b**) ESI negative mode with 1286 datapoints.

Based on these ionization efficiencies, predictive models for positive and negative mode were developed. The overall root mean squared prediction error was 2.2 times (training set 1.9 and test set 3.0 times) (Fig. 2a and Table S11). This means that if the ionization efficiency of compound A is predicted to be 100 times higher than the ionization efficiency of the methyl benzoate the actual ionization efficiency would be 45 to 220 higher than that of methyl benzoate (log*IE* = 2.00 ± 0.34).

In negative mode, the best performing model was obtained also with random forest regression with 145 significant descriptors and 100 regression trees. The regression model explained 93% of the variation in ionization efficiency values. The overall root mean squared error was 2.0 times (training set 2.0 and test set 2.3 times, Fig. 2b).

Upon closer examination of the ionization efficiency prediction model in ESI positive mode, it was observed that the model performed universally well for different organic modifier percentages (Fig. S5). The lowest prediction error, 1.4 times, was observed for eluents containing 20% of organic modifier and the highest prediction error, 1.9 times, for eluent containing 90% organic modifier. This is expected, as eluents containing 20% of organic modifier had the highest number of data points, which improved prediction accuracy. Additionally, the model was well-performing for both methanol as well as for acetonitrile containing eluent compositions (Figs. S5 and S6). Based on the pH of the eluent, basic conditions had the highest prediction error; 2.5 times and 3.7 times for the training and test set, respectively.

Similar trends were observed for ESI negative mode; the prediction accuracy for the pure organic modifier was the lowest (prediction error of 4.1 times, Figs. S7 and S8). Regarding the pH, no significant differences in the prediction accuracy were observed (Fig. S7).

The most influential parameters (Table S12) were a number of structural parameters and also solvent parameters. PaDEL descriptors used in the Random Forest regression are 2D structural parameters and are, therefore, not directly linked to classical physicochemical parameters; however, some of the most influential parameters can be interpreted. From structure-related parameters, number of hydrogen atoms and number of nitrogen atoms was important in the model in ESI positive mode. The number of hydrogen atoms is associated with the size and hydrophobicity of the compounds while the number of nitrogen atoms is associated with the basicity of the compound. Also, the mobile phase parameters pH, viscosity, and presence of ammonium ions in the mobile phase were significant. Previous studies have shown that these factors can influence the response of compounds by orders of magnitude^18–20^. Here, the model confirms the previous empirical findings.

### Compatibility between different instruments

**Figure 3:**
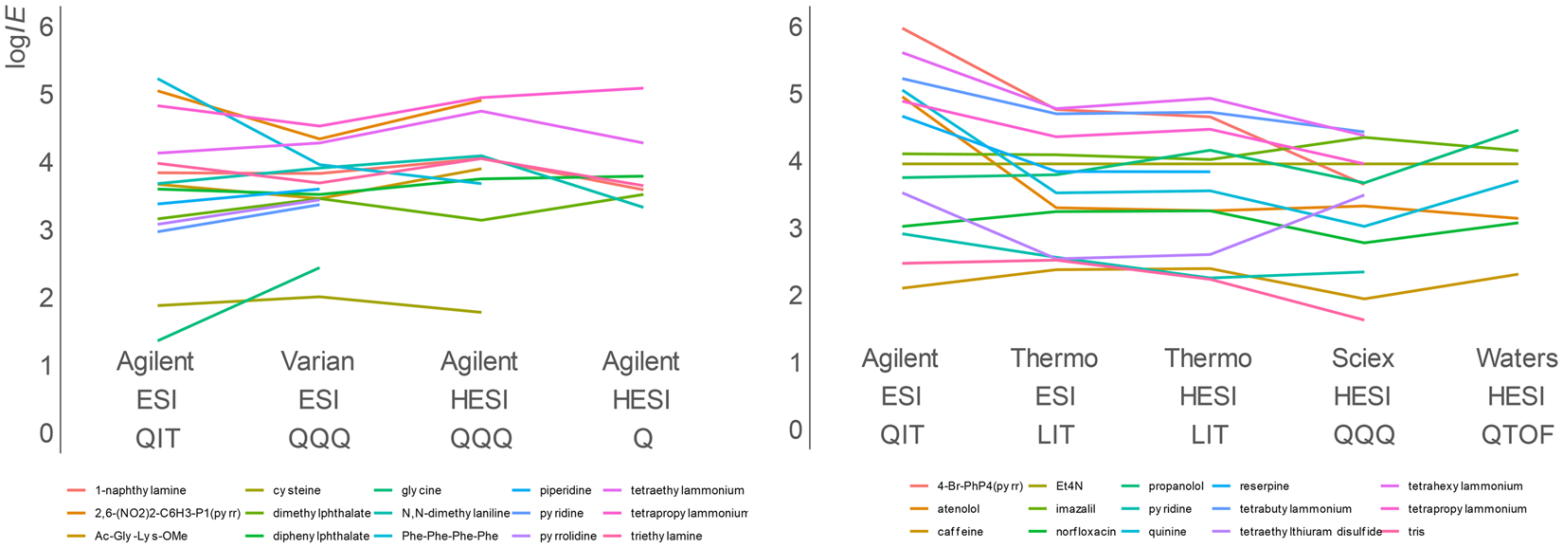
Measured ionization efficiencies on different instruments in ESI positive mode based on two subsets of druglike compounds measured on different instruments in the same eluent composition (acetonitrile/0.1% formic acid(aq) 80/20). Full data are shown in Table S2. ESI denotes conventional pneumatically assisted electrospray ionization source, HESI heated electrospray ionization source, QIT quadrupole ion trap, QQQ triple quadrupole, LIT linear iontrap, QTOF quadrupole time-of flight. Left and right correspond to two datasets.

It is known that a compound’s response varies from one LC/MS instrument to another; however, we have previously shown that the relative log*IE* values measured on different instruments are in good correlation to each other (Figs. 3 and S9)^22,23^. The ionization efficiency model was primarily developed on an ion trap instrument; however, for this study, we expanded the types of LC/MS instruments used for measuring ionization efficiency values. Altogether seven instruments and 9 different instrument-ionization source combinations (Table S8) and different mass analyser types from labs around the world have been included in studying the ionization efficiency. On all instruments, generic default settings have been used. It is known that some instruments (e.g. QQQ) may have lower mass cut-offs. Here, these effects are incorporated in the agreement of ionization efficiency values.

The comparison of the ionization efficiency values for compounds measured on a number of instruments reveals good intra-instrumental consistency (Figs. 3 and S9). The compounds being highest responders on one instrument are also generally top performers on the other instruments. Still, some instruments compress the ionization efficiency values more than other instruments. However, these effects could not be associated with a known lower mass cut-off of mass analysers or ionization source type (ESI vs HESI). Therefore, a more complicated relationship between the source design and ion optics design is likely to be important.

At the same time, it is not reasonable to build time and data-intensive prediction algorithms on each instrument. Therefore, it is necessary to have a robust approach to transfer the predicted values between different instruments. In order to achieve this, we use a linear correlation observed between the ionization efficiency values measured on different instruments (see SI for more details).

The feasibility of using linear correlation for transferring the predicted ionization efficiency values can be estimated by comparing the prediction error of ionization efficiency values for different instruments. We observed a good consistency between the prediction errors from instrument to instrument (Fig. S10). The highest average prediction error was 2.8 times (Waters Synapt G.2 Z-spray) and the lowest was 1.5 times (Thermo LTQ ESI). Both of these values are close to the repeatability of the log*IE* values. Therefore, it can be concluded that ionization efficiency values can be transferred between instruments with reasonable accuracy.

Additionally, we developed a model to predict ionization efficiency values with PaDEL descriptors and random forest regression based on data from a single instrument. For this, we chose data from an Agilent XCT instrument, which had been used to measure the largest number of ionization efficiency values (1816 data points). The mean prediction error for a single instrument-based model (ESI + mode) was 1.8 and 3.0 times for training and test set, respectively. The achieved prediction error values for a single instrument-based model were not significantly different from the generic model incorporating data from seven different instruments. Therefore, the developed model is robust for variations caused by different instruments and can be applied to predict ionization efficiency values measured on different mass spectrometers.

### Validation: ionization efficiency and quantification of pesticides and mycotoxins in cereals

The proposed was validated based on 35 pesticides and mycotoxins (Table S7) measured under gradient separation on a triple quadrupole instrument. The list included 28 compounds not included in the training or test set. The log*IE* values were measured from the analysis of standard solutions of the 35 validation compounds and the values range from 1.60 to 4.11 with the average of 3.10 and median 3.29. The predicted log*IE* values take into account the eluent composition at retention time. The log*IE* prediction with the random forest model showed reasonable accuracy (RMSE 0.64, Fig. S11).

The predicted ionization efficiency was further used to predict the concentrations of the compounds detected assuming that the structure is known. For this, the compounds were spiked into blank oat, barley, rye, wheat, rice, and maize and analysed. For each compound, the ionization efficiency was predicted in ESI positive mode. To transform the predicted ionization efficiencies to instrument-specific response factors, a set of 31 compounds (Table S7) was used. Thereafter, the instrument-specific response factors were used to convert LC/MS signals into concentration (Eq.).

7 $$c=\frac{Signal}{{10}^{\log R{F}_{pred}}}$$

**Figure 4:**
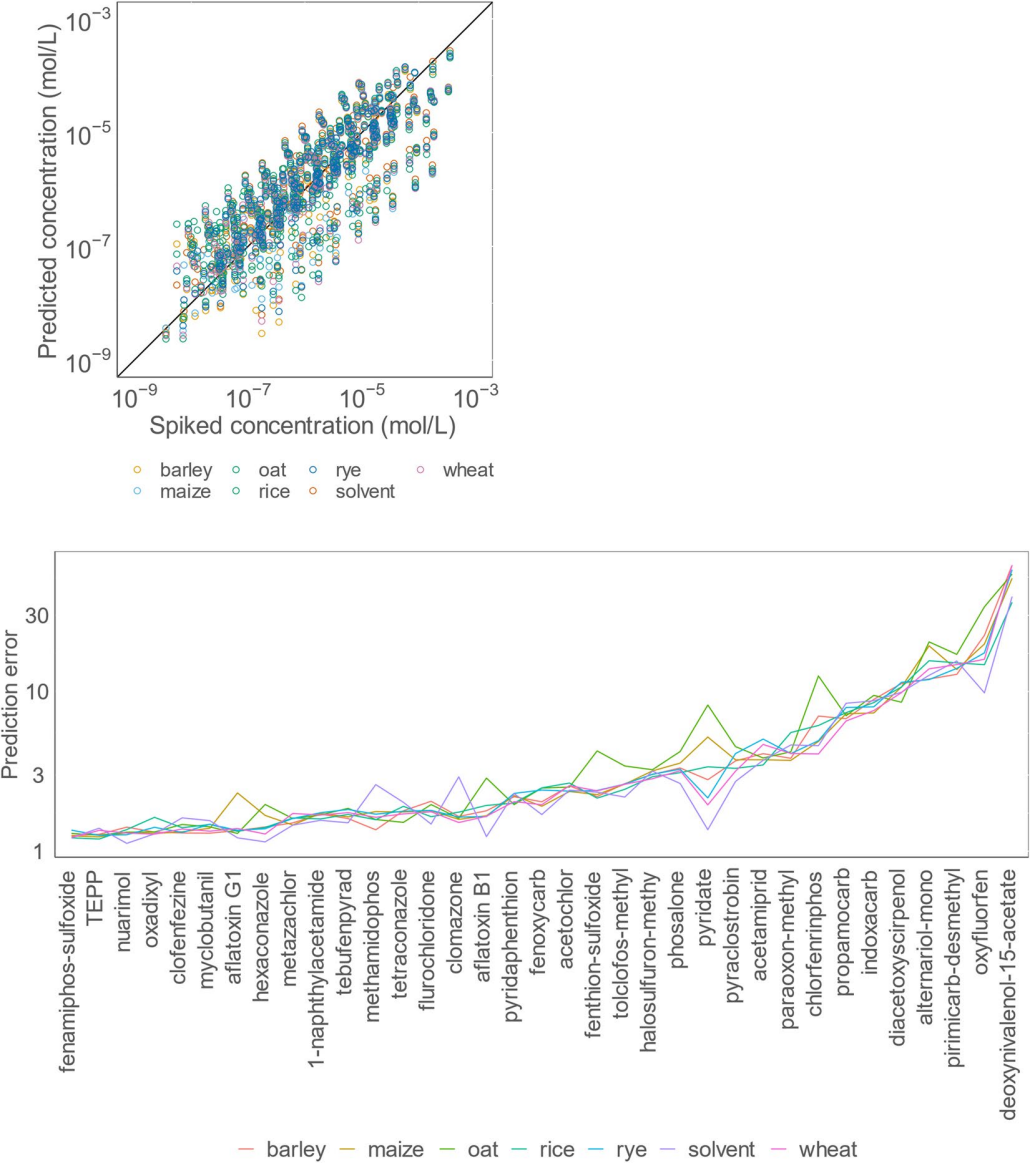
Performance of concentration prediction in the example of pesticides in cereals. above: concentration prediction of pesticides in cereal samples. below: prediction error of pesticide concentration in cereal samples, y-axis in logarithmic scale.

On average, the concentrations were predicted with the prediction error of 5.4 times (Fig. 4a, compound separated graphs on Fig. S12, Table S13). This means that if the pesticide concentration is estimated to be 1 ppm it would actually lie between 0.2 and 6 ppm. Compared to the conventional approach of assuming the equal response to all compounds detected (average prediction error of 526 times, Table S14), the developed approach improved prediction accuracy around 10 times and significantly reduces the width of the confidence interval. Consequently, more effective decision making can be made based on the predicted concentrations.

The lowest error observed was 1.2 times for TEPP in rice matrix, while the largest error, 62 times was observed for deoxynivalenol-15-acetate in barley matrix. For 88% of the compounds, the prediction error was lower than 10 times, for 76% of the compounds it was lower than 5 times, and for 48% of the compounds, it was lower than 2 times. The three compounds with the highest prediction error were deoxynivalenol-15-acetate (52 times), oxyfluorfen (19 times), and pirimicarb-desmethyl (15 times). The three best-performing compounds are fenamiphos-sulfoxide (1.2 times), TEPP (1.3 times), and nuarimol (1.3 times). These prediction errors are especially promising for mycotoxins for which the standard substances are either rarely available or very expensive. The large errors could not be associated with specific compound properties.

Additionally, we tested sets of 15, 10 and 6 compounds for transforming the predicted ionization efficiency values to instrument-specific response factors. For this random sets of 15, 10 or 6 compounds were sampled from the 31 compounds. On average, sets of 15, 10, and 6 compounds resulted in concentration prediction errors of 5.0, 5.0, and 5.0 times, respectively, which were not significantly different from the average prediction error observed for 31 compounds. Therefore, comparable prediction accuracies can be achieved with a smaller set of compounds. However, it needs to be considered that the compounds used should possess a wide range of chemical properties as well as a wide range of ionization efficiency values to allow for the establishment of a reasonable regression approach. Additionally, uniform elution distribution during the chromatographic run is beneficial.

### Different matrices

Any model that aims at providing quantitative information needs to be applicable in a variety of matrices in order to be truly useful to researchers. The importance of matrices with LC/MS is even more relevant than for other analytical techniques, due to the possibility of significant matrix effects in the ESI source. A matrix effect is the suppression of ionization of a compound due to co-eluting compound(s). Previously, it has been qualitatively observed that the matrix effect and ionization efficiencies are influenced by the physicochemical properties of the compound^40^. Therefore, while applying the ionization efficiency predictions for concentration estimations, it was assumed that a model that considers ionization efficiencies also helps to account for matrix effect. This assumption was based on the fact that a small set of compounds with known concentrations were spiked into every sample; this helped to account for the differences arising from the instrument and also for matrix effects (Eq. 7).

Regarding different matrices, the prediction accuracy for all cereals was very similar. The lowest prediction error was observed for wheat and rice, 4.8 times, and highest for oat, 6.3 times (Table S15). This is expected, as oat samples possess a high content of polar lipids and free fatty acids^41^ which possess high surface affinity and are, therefore, expected to cause ionization suppression^42^. Also, the mean prediction error for solvent (average 4.6 times) and all studied cereals (5.5 times) was comparable.

Moreover, the matrix effect is expected to vary strongly from sample to sample even for the same food commodity. The compounds that performed worst in one matrix performed also performed poorly in other matrices, and the best performers were among the top for all matrices (Fig. 4b). The consistency of the prediction error form one matrix to another indicates the insignificance of matrix effect and strongly indicates that the developed approach using ionization efficiency predictions, together with the transformation, helped to account for matrix effects.

### Application area

The application range of all models depends on the data that have been used to train the model. The compounds covered by the model include protonated [M+H]^+^ and intrinsically charged compounds [M]^+^ in positive mode and deprotonated [M-H]^−^compounds in the negative mode. This defines the limitations of the application range of the model. For examples, compounds forming primarily sodium or ammonium adducts and no protonated ions are not within the scope of this approach.

We were additionally interested in comparing the compounds used in this study to compounds detectable with LC/MS based non-targeted screening. For this purpose, we compared the properties of the compounds used in this study with some of the most common databases; namely, NORMAN database^43^, HMDB^9^, and DrugBank^44^. NORMAN database is a database compiled by NORMAN network members and contains compounds that are important for various application areas from solvents to industrial chemicals, personal care products to metabolites. HMDB contains information about metabolites with different origins and DrugBank contains approved small molecule drugs, approved biologics (proteins, peptides, vaccines, and allergenics), nutraceuticals and experimental (discovery-phase) drugs. It is important to consider that LC/MS is unable to analyse all of these compounds due to limitations in retention and ionization. For HMDB and NORMAN it was possible to extract only the compounds that had been analysed with LC/MS. The compounds included in this study were compared with the databases based on the principal component analysis of PaDEL descriptors. It was observed that compounds included in this study covered a very large part on the chemical space of interest in HMDB and NOMRAN (Figs. S2 and S3). The coverage was somewhat less for DrugBank. This was expected, as the DrugBank includes also large biomolecules out of the scope of the model. Therefore, it is important to keep in mind the limitations of the initial data used in the modelling while applying the model and we hope to widen the model application are further in the future by incorporating (1) compounds forming adducts and (2) larger compounds, such as peptides.

## Discussion

For the case study presented here, quantifying a variety of pesticides and mycotoxins in cereal matrices, we have shown that ionization efficiencies can be used to improve the accuracy of concentration predictions for small molecules that lack standard substances. In general, to obtain a reliable model, it is suggested to include several compounds that have been analysed together with the sample and quantified. Such compounds could be compounds confirmed with the aid of standard substances or compounds from quality control samples. This makes full scan LC/HRMS extremely appealing, as a combined targeted and non-targeted analysis method can be used.

Additionally, for risk assessment of contaminants in food and environmental samples, an estimated concentration of a compound is required to evaluate the potential risk of the exposure. In most cases, the error associated with other parts of the risk assessment (like intake and toxicology) is even larger than the error in concentration prediction^45^. Therefore, the ionization efficiency based quantification approach has high potential to complement non-targeted analysis and aid decision making based on the analysis results. The tool is made available at app.quantem.co. The approach has been developed on several instruments and validated on the example of five complicated matrices. Therefore, the quantification accuracy also represents the effects arising from mass discrimination and matrix effect. Validation for other applications is though, desirable.

## Supplementary information

Supporting information.

Table S2.

Table S5.

Table S17.

Table S16.

## Data Availability

All data used for model development and validation are available as supporting information.
