# Supplementary material for: Quantification for non-targeted LC/MS screening without standard substances
Source: Sci Rep. 2020 Apr 2;10:5808. doi: 10.1038/s41598-020-62573-z (PMC7118164; doi:10.1038/s41598-020-62573-z)
Supplement: Supplementary file 1 — Supporting information. [file 41598_2020_62573_MOESM1_ESM.pdf]

Quantification for non-targeted LC/MS screening without standard substances

Jaanus Liigand[1], Tingting Wang[2], Joshua J. Kellogg[3], Jørn Smedsgaard[2], Nadja B. Cech[3], Anneli Kruve[1,4]

[1] Institute of Chemistry, Faculty of Science and Technology, University of Tartu, Ravila 14A, 50411, Tartu, Estonia

[2] National Food Institute, Research Group for Analytical Food Chemistry, Technical University of Denmark, Kemitorvet Building 202, Kgs. Lyngby, DK-2800, Denmark

[3] Department of Chemistry & Biochemistry, University of North Carolina at Greensboro, Greensboro, North Carolina 27412, United States

[4] Department of Environmental Science and Analytical Chemistry, Stockholm University, Svante Arrhenius väg 16, 106 91 Stockholm

Table S1 In literature available studies focusing on ionization efficiency investigation and modelling. 5
Table S2 log*IE* values collected from previous studies and measured in this study. 9
Table S3 The eluent compositions used for model development in ESI positive mode. 10
Table S4 The eluent compositions used for model development in ESI negative mode. 15
Table S5 Classifications of studied compounds using ClassyFire. 17
Table S6 The most prominent superclasses covered by the compounds included in this study. Classifications of studied compounds using ClassyFire. 17
Table S7 Compounds used in validation and application study. 18
Table S8 Instruments used to study ionization efficiencies in this study. 21
Table S9 Significant descriptors in ESI positive mode model. 22
Table S10 Significant descriptors in ESI negative mode model. 24
Table S11 Ionization efficiency prediction model characteristics: absolute error distribution in log*IE* units and times differences. 25
Table S12 TOP 15 most important features in developed models in ESI positive and negative mode. 26
Table S13 Absolute concentration prediction error characteristics based on the validation set. 27
Table S14Comparison of errors in concentration prediction by compound in case of pesticides and mycotoxins in cereal matrices using three different approaches. 28
Table S15Comparison of errors in concentration prediction by matrix in case of pesticides and mycotoxins in cereal matrices using ionization efficiency prediction. 30
Table S16 Properties of pesticides studied in application study. 31
Table S17 Raw data collected in application study in example of pesticides and mycotoxins in cereals. 31
Table S18 Summary of data used for model development and concentration prediction. 32

Figure S1 A comparison of the chemical space coverage based on logP values. 33
Figure S2 A comparison of the chemical space covered by compounds included in this study (training, test, and validation sets) in comparison to (left) NORMAN, (middle) HMDB, and (right) DrugBank databases. 34
Figure S3 The PCA analysis of the compounds from training, test and validation set based on the PaDEL descriptors. 35
Figure S4 PCA analysis of the training dataset compounds(n = 353). 36
Figure S5 Comparison of prediction errors ionization efficiencies between acetonitrile and methanol containing solvents in ESI positive mode. 38
Figure S6 Comparison of the prediction error of ionization efficiencies between neat acetonitrile and methanol in ESI positive mode. 39
Figure S7 Comparison of the prediction error of ionization efficiencies in acetonitrile containing solvents in ESI negative mode. 40
Figure S8 Comparison of the prediction error of ionization efficiency between different solvents in ESI negative mode. 41
Figure S9 Correlation between logIE values measured for set of compounds in the same eluent composition on different mass spectrometric setups. 42

Figure S10 Comparison of the prediction error of ionization efficiency values for different instruments (Table S7) in ESI positive mode. 44
Figure S11 The predicted ionization efficiency values for the validation compounds relative to the measured values. 45
Figure S12 Comparison of predicted and spiked concentration in case of pesticides in cereal samples. 46
Figure S13 Comparison of the prediction error of ionization efficiencies for different ionization efficiency groups in ESI positive mode. 47
Figure S14 Comparison of the prediction error of ionization efficiencies for different ionization efficiency groups in ESI negative mode. 48
Code S1 Code used for model development. 489

Methods

Eluent parameters

The viscosity of organic modifier water binary mixture[1–3] was calculated using the general model:

$$viscosity\ (mPa \cdot s) = A \cdot (org\% \cdot 100)^2 + B \cdot org\% \cdot 100 + C \qquad \text{Eq. 1}$$

The surface tension of organic modifier water binary mixture[4,5] was calculated using the general model:

$$surface\ tension\ (\mathrm{mN/m}) = \sigma_1 + D \cdot \sigma_1 \cdot org\% + (E \cdot \sigma_2 - D \cdot \sigma_1 - \sigma_1) \cdot org\%^2 + (\sigma_2 - D \cdot \sigma_2) \cdot org\%^3 \qquad \text{Eq. 2}$$

Polarity index of organic modifier water binary mixture[6] was calculated using the general model:

$$polarity\ index = F \cdot org\% + G \cdot (1 - org\%) \qquad \text{Eq. 3}$$

NH_4^+ presence parameter was 1 if the additive included either ammonia or ammonium salt (ammonium acetate, ammonium formate, ammonium bicarbonate, ammonium fluoride), otherwise, it was kept 0.

Parameters used to calculate eluent descriptors.

name	A	B	C	D	E	F	G	σ_1	σ_2
**acetonitrile**	$-1.04\cdot10^{-4}$	$4.36\cdot10^{-3}$	$8.84\cdot10^{-1}$	-2.9	7.14	5.8	–	–	27.9
**methanol**	$-3.59\cdot10^{-4}$	$3.20\cdot10^{-2}$	$9.03\cdot10^{-1}$	-2.2	5.62	5.1	–	–	22.1
**isopropanol**	$-4.74\cdot10^{-4}$	$5.89\cdot10^{-2}$	$7.88\cdot10^{-1}$	-3.9	15.6	3.9	–	–	17
**acetone**	$-3.13\cdot10^{-4}$	$2.47\cdot10^{-2}$	$9.02\cdot10^{-1}$	-2.5	6.84	5.1	–	–	22.2
**water**	–	–	–	–	–	–	10	71.8	–

Data unification between different instruments

To develop the model, log*IE* values measured on different instruments were transformed to unified log*IE* values. The unification was performed using the intersection of compounds measured on two instruments in the eluent composition acetonitrile/0.1% formic acid (aq) 80/20. A calibration graph was constructed logIE (Instrumentn) vs log*IE* (Agilent XCT). The log*IE* values measured on different instrument than Agilent XCT ion trap were transformed as follows:

$$\log IE\ (Agilent\ XCT) = slope \cdot \log IE\ (Instrument_n) + intercept \qquad \text{Eq. 4}$$

Table S1 In literature available studies focusing on ionization efficiency investigation and modelling.

Ref.	Compound type	Number of unique compounds	Eluent	Mode	Model	Matrix
[7]	drug like	10	0.1 % formic acid/acetonitrile 60/40	+	no	solvent
[8]	steroids	30	100 % methanol; ammonia (pH = 10)/methanol 50/50	+/−	PLS	solvent
[9]	oligonucleotide	11	water/methanol 50/50 with ion-pairing reagents	−	PLS	solvent
[10]	metabolites	9	0.5 mM ammonium acetate/methanol 1/9	+	no	urine
[11]	natural products	8	0.1 % formic acid/acetonitrile	+	no	solvent
[12]	lipids	15	100 % methanol	+	no	solvent
[13]	drugs	170	1 mM ammonium acetate/acetonitrile 50/50	+	PCA, Projection Pursuit. Classification and Regression Trees. PLS and Stepwise MLR	solvent
[14,15]	oligopeptides	23	0.5 % acetic acid/methanol 50/50	+	no	solvent
[16]	metabolites	58	0.1 % formic acid/methanol 50/50	+	MLR	solvent; RBC lysates

Ref.	Compound type	Number of unique compounds	Eluent	Mode	Model	Matrix
17	lipids	8	5 mM ammonium acetate/acetonitrile	+	no	NIST human plasma; egg yolk; porcine liver
18	drugs	77	0.1 % formic acid/acetonitrile	+	MLR	solvent
19	drugs	40	0.1 % acetic/acetonitrile (0.1 % acetic)	+	no	solvent
20	druglike	19	0.5 % acetic acid in methanol	+	no	solvent
21	druglike	20	7 mM acetic acid /methanol 20/80; 7 mM ammonia/methanol 20/80	+/−	ANN	solvent
22	amino acids	4		+	no	no
23	natural products	25	10 mM ammonium formate/acetonitrile 60/40; 50/50; 40/60; 30/70; 20/80	− (formate adduct)	no	solvent, *S. habrochaites* leaves
24	drugs	110	water/acetonitrile 20/80	−	PLS	solvent
25	sartans	7	pH = 5.5/methanol 40/60	+	ANN	solvent
26	drugs	71	water/acetonitrile 50/50	+	no	solvent
27	phenols	35	100 % methanol; 100 % acetonitrile; methanol/water 50/50; acetonitrile/water 50/50	−	no	solvent
28	amino acids. derivates	127	acetonitrile (0.1 % formic acid)/0.1 % formic acid	+	MLR	solvent
29	druglike	49	100 % acetonitrile; 100 % acetone;	−	no	solvent

Ref.	Compound type	Number of unique compounds	Eluent	Mode	Model	Matrix
			100 % methanol;<br>100 % water			
30	natural products	13	5 mM ammonium acetate/ methanol (5 mM ammonium acetate)	−	no	olive oil
31	lipids	5	tetrahydrofuran	−	MLR	kerogen extract
32	small bases	56	pH = 3/acetonitrile 50/50; 20/80;<br>pH = 7/acetonitrile 50/50; 20/80	+	no	solvent
33	lipids	15	chloroform/methanol 1:2<br>1 % ammonia	−	no	solvent
34	peptide	4	0.1 % ammonium acetate/acetonitrile<br>0.5 % acetic acid 0.1 % ammonium acetate/acetonitrile	+	no	solvent
35	drug	99	ammonia (pH = 10)/methanol 50/50	+	PLS	solvent
36	glycans	8	1 mM NaOH/methanol 50/50	+	no	*Drosophila melanogaster*. solvent
37	steroid	7	10 mM ammonium acetate/acetonitrile/isopropanol (9/1);<br>0.02 % acetic acid/ acetonitrile/isopropanol 9/1	−	no	solvent;<br>human plasma
38	natural products	4	water	+	MLR	secondary organic

Ref.	Compound type	Number of unique compounds	Eluent	Mode	Model	Matrix
						aerosol in water
39	small molecules	17	pH= 3.1 /acetonitrile	−/+	no	solvent
40	oligopeptide	12	1 mM ammonium acetate (pH = 6)/methanol 90/10; 1 mM ammonium acetate (pH = 10)/methanol 90/10	+	MLR, regression tree, SVR	solvent
41	glycans	13	0.1 % formic acid/acetonitrile 65/35	+	no	tryptic BSA peptides
42	lipophilic marine algal toxins	10	2 mM ammonium formate and 50 mM formic acid/acetonitrile (with additive) 40/60	+/−	no	solvent; mussel
43	flavonoids	6	formic acid acid (pH = 3)/acetonitrile	−	no	solvent
44	lipids	34	10 mM ammonium formate in water/acetonitrile (6/4) and 10 mM ammonium formate isopropanol/acetonitrile (9/1)	+/−	no	human plasma; mouse brain tissue; Jurkat cell pellet
45	small acids	25	0.025 % acetic acid/methanol	−	MLR	solvent
46	amino acids	17	2 % acetic acid/methanol 50/50	+	no	solvent
47	flavonoids	19	0.1 % formic acid/acetonitrile	−	no	extract of *G. biloba*

Table S2 log*IE* values collected from previous studies and measured in this study.

See logIE_values.xlsx

Table S3 The eluent compositions used for model development in ESI positive mode (* denotes measurements carried out during this study).

Organic modifier percentage	Organic modifier	Water phase percentage	Additive	Additive concentration (mM)	pH(aq)	Number of unique compounds	Ref.
100	Methanol	0	formic acid	27.0	2.7	35	*,[2]
100	Methanol	0	trifluoroacetic acid	5.0	3.6	11	[2]
100	Methanol	0	oxalic acid	5.0	4.1	11	[2]
100	Methanol	0	oxalic acid	1.0	5.4	11	[2]
100	Methanol	0	formic acid	5.0	5.4	11	[2]
100	Methanol	0	formic acid	1.0	5.9	11	[2]
100	Methanol	0	formic acid ammonium acetate	5.0	6.1	11	[2]
100	Methanol	0	formic acid ammonium acetate	5.0	6.3	11	[2]
100	Methanol	0	acetic acid	5.0	6.5	11	[2]
100	Methanol	0	formic acid	0.1	6.6	11	[2]
100	Methanol	0	formic acid ammonium acetate	5.0	7.6	11	[2]
100	Methanol	0	formic acid ammonium acetate	5.0	8.4	11	[2]
100	Methanol	0	ammonium formate	5.0	9.4	11	[2]
100	Methanol	0	ammonium acetate	5.0	10.1	11	[2]
100	Methanol	0	ammonia	5.0	12.2	11	[2]
90	Methanol	10	oxalic acid	1.0	2.1	20	[3]
90	Methanol	10	formic acid	3.0	2.7	20	[3]
90	Methanol	10	formic acid	1.0	2.9	20	[3]
90	Methanol	10	propionic acid	1.0	3.4	20	[3]
80	Methanol	20	formic acid	27.0	2.7	89	*
50	Methanol	50	trifluoroacetic acid	10.0	2.0	40	*
50	Methanol	50	formic acid	27.0	2.7	40	*
50	Methanol	50	ammonium fluoride	10.0	5.5	40	*

Organic modifier percentage	Organic modifier	Water phase percentage	Additive	Additive concentration (mM)	pH(aq)	Number of unique compounds	Ref.
50	Methanol	50	ammonium acetate	10.0	6.8	40	*
50	Methanol	50	ammonium formate	10.0	6.8	40	*
50	Methanol	50	ammonium bicarbonate	10.0	7.8	40	*
50	Methanol	50	ammonia	52.0	10.7	36	*
20	Methanol	80	formic acid	27.0	2.7	40	*
100	Acetonitrile	0	formic acid	27.0	2.7	35	*
90	Acetonitrile	10	oxalic acid	1.0	2.1	20	[3]
90	Acetonitrile	10	formic acid	1.0	2.9	20	[3]
90	Acetonitrile	10	propionic acid	1.0	3.4	20	[3]
80	Acetonitrile	20	trifluoroacetic acid	3.0	1.9	65	[4]
80	Acetonitrile	20	oxalic acid	1.0	2.1	17	[5]
80	Acetonitrile	20	formic acid	5.0	2.7	352	*, [3,6–9]
80	Acetonitrile	20	ammonium acetate	1.0	3.0	20	[4]
80	Acetonitrile	20	acetic acid	3.0	3.2	17	[5]
80	Acetonitrile	20	ammonium acetate	1.0	3.5	20	[4]
80	Acetonitrile	20	ammonium acetate	1.0	4.0	20	[4]
80	Acetonitrile	20	ammonium acetate	1.0	4.5	19	[4]
80	Acetonitrile	20	ammonium acetate	1.0	5.0	26	[4,7]
80	Acetonitrile	20	ammonium acetate	1.0	5.5	20	[4]
80	Acetonitrile	20	–	–	–	16	[5]
80	Acetonitrile	20	ammonium acetate	1.0	6.0	20	[4]
80	Acetonitrile	20	ammonium acetate	1.0	6.5	20	[4]
80	Acetonitrile	20	ammonium acetate	1.0	6.7	15	[4]
80	Acetonitrile	20	ammonium acetate	1.0	7.0	46	[4]

Organic modifier percentage	Organic modifier	Water phase percentage	Additive	Additive concentration (mM)	pH(aq)	Number of unique compounds	Ref.
80	Acetonitrile	20	methylamine	0.0	7.9	14	[5]
80	Acetonitrile	20	ammonia	0.2	9.8	10	[7]
80	Acetonitrile	20	ammonia	10.0	10.7	36	[7]
50	Acetonitrile	50	trifluoroacetic acid	10.0	2.0	40	*
50	Acetonitrile	50	formic acid	27.0	2.7	45	*,[7]
50	Acetonitrile	50	ammonium acetate	2.5	5.0	10	[7]
50	Acetonitrile	50	ammonium fluoride	10.0	5.5	40	*
50	Acetonitrile	50	ammonium acetate	10.0	6.8	40	*
50	Acetonitrile	50	ammonium formate	10.0	6.8	40	*
50	Acetonitrile	50	ammonium bicarbonate	10.0	7.8	40	*
50	Acetonitrile	50	ammonia	0.5	9.8	10	[7]
50	Acetonitrile	50	ammonia	52.0	10.7	50	*
20	Acetonitrile	80	trifluoroacetic acid	10.0	1.9	28	[4]
20	Acetonitrile	80	formic acid	27.0	2.7	60	*,[7]
20	Acetonitrile	80	ammonium acetate	4.0	3.0	17	[4]
20	Acetonitrile	80	ammonium acetate	4.0	3.5	21	[4]
20	Acetonitrile	80	ammonium acetate	4.0	4.0	20	[4]
20	Acetonitrile	80	ammonium acetate	4.0	4.5	19	[4]
20	Acetonitrile	80	ammonium acetate	4.0	5.0	20	[4]
20	Acetonitrile	80	ammonium acetate	4.0	5.5	19	[4]
20	Acetonitrile	80	ammonium acetate	4.0	6.0	20	[4]
20	Acetonitrile	80	ammonium acetate	4.0	6.5	25	[4]
20	Acetonitrile	80	ammonium acetate	4.0	7.0	19	[4]

Organic modifier percentage	Organic modifier	Water phase percentage	Additive	Additive concentration (mM)	pH(aq)	Number of unique compounds	Ref.
90	Isopropanol	10	oxalic acid	1.0	2.1	20	[3]
90	Isopropanol	10	formic acid	1.0	2.9	18	[3]
90	Isopropanol	10	propionic acid	1.0	3.4	14	[3]
90	Acetone	10	oxalic acid	1.0	2.1	19	[3]
90	Acetone	10	formic acid	1.0	2.9	19	[3]
90	Acetone	10	propionic acid	1.0	3.4	20	[3]
0	–	100	oxalic acid	50.0	1.4	11	[2]
0	–	100	oxalic acid	5.0	2.1	11	[2]
0	–	100	trifluoroacetic acid	5.0	2.4	11	[2]
0	–	100	citric acid	5.0	2.5	11	[2]
0	–	100	formic acid	27.0	2.7	39	*,[2]
0	–	100	oxalic acid	1.0	2.8	11	[2]
0	–	100	formic acid	5.0	2.8	11	[2]
0	–	100	formic acid ammonium acetate	5.0	3.0	11	[2]
0	–	100	formic acid. ammonium formate	5.0	3.0	11	[2]
0	–	100	formic acid	1.0	3.1	11	[2]
0	–	100	acetic acid	5.0	3.2	11	[2]
0	–	100	formic acid ammonium acetate	5.0	3.3	11	[2]
0	–	100	formic acid ammonium acetate	5.0	3.5	11	[2]
0	–	100	formic acid	0.1	3.7	11	[2]
0	–	100	formic acid ammonium acetate	5.0	4.0	11	[2]
0	–	100	formic acid. ammonium formate	5.0	4.0	11	[2]
0	–	100	formic acid ammonium acetate	5.0	5.0	11	[2]

Organic modifier percentage	Organic modifier	Water phase percentage	Additive	Additive concentration (mM)	pH(aq)	Number of unique compounds	Ref.
0	–	100	formic acid ammonium acetate	5.0	5.0	11	[2]
0	–	100	ammonium formate	5.0	5.5	11	[2]
0	–	100	formic acid ammonium acetate	5.0	5.9	11	[2]
0	–	100	formic acid ammonium formate	5.0	5.9	11	[2]
0	–	100	ammonium acetate	1.0	6.1	11	[2]
0	–	100	ammonium acetate	5.0	6.7	11	[2]
0	–	100	ammonium acetate	10.0	6.8	11	[2]
0	–	100	methylamine	1.0	7.9	9	[2]
0	–	100	ammonia ammonium acetate	5.0	8.1	11	[2]
0	–	100	ammonia ammonium acetate	5.0	9.0	11	[2]
0	–	100	ammonia	1.0	9.4	11	[2]
0	–	100	ammonia	5.0	10.1	11	[2]
0	–	100	ammonia	10.0	10.3	11	[2]

Table S4 The eluent compositions used for model development in ESI negative mode.

Organic modifier percentage	Organic modifier	water phase percentage	Additive	additive concentration (mM)	pH(aq)	Number of unique compounds	Ref.
100	Methanol	0	ammonia	52.0	10.5	17	*
100	Methanol	0	-	-	7.0	17	*
80	Methanol	20	ammonia	10.0	10.5	17	*
80	Methanol	20	-	-	7.0	16	*
60	Methanol	40	ammonia	21.0	10.5	17	*
60	Methanol	40	-	-	7.0	17	*
40	Methanol	60	ammonia	31.0	10.5	17	*
40	Methanol	60	-	-	7.0	17	*
20	Methanol	80	ammonia	41.0	10.5	17	*
20	Methanol	80	-	-	7.0	17	*
0	Methanol	100	-	-	7.0	17	*
100	Acetonitrile	0	ammonia	52.0	10.5	60	[10]
100	Acetonitrile	0	-	-	7.0	17	[11]
80	Acetonitrile	20	ammonia	10.0	10.5	101	*,[8,11–13]
80	Acetonitrile	20	ammonium acetate	1.0	7.8	59	[14]
80	Acetonitrile	20	ammonium acetate	1.0	7.0	22	*
80	Acetonitrile	20	-	-	7.0	16	[11]
80	Acetonitrile	20	ammonium acetate	0.2	5.0	60	[14]
80	Acetonitrile	20	ammonium acetate	1.0	5.0	62	[14]
80	Acetonitrile	20	ammonium acetate	1.0	3.5	56	[14]
80	Acetonitrile	20	formic acid	5.0	2.8	61	[14]
60	Acetonitrile	40	ammonia	21.0	10.5	17	[11]
60	Acetonitrile	40	-	-	7.0	17	[11]
50	Acetonitrile	50	ammonia	26.0	10.5	59	[14]
40	Acetonitrile	60	ammonia	31.0	10.5	17	[11]
40	Acetonitrile	60	-	-	7.0	17	[11]
20	Acetonitrile	80	ammonia	41.0	10.5	62	[14]
20	Acetonitrile	80	-	-	7.0	17	[11]
20	Acetonitrile	80	ammonium acetate	4.0	5.0	57	[14]
20	Acetonitrile	80	formic acid	21.0	2.8	49	[14]
0	-	100	ammonia	52.0	10.5	17	[11]

Organic modifier percentage	Organic modifier	water phase percentage	Additive	additive concentration (mM)	pH(aq)	Number of unique compounds	Ref.
0	-	100	ammonia	52.0	10.5	17	[11]
0	-	100	-	-	7.0	17	[11]

Table S5 Classifications of studied compounds using ClassyFire.[48]

See classyfire_classification.csv

Table S6 The most prominent superclasses covered by the compounds included in this study. Classifications of studied compounds using ClassyFire.[48]

Superclass	n
Benzenoids	163
Organoheterocyclic compounds	83
Organic acids and derivatives	54
Organic nitrogen compounds	28
Lipids and lipid-like molecules	16
Organic oxygen compounds	9
Phenylpropanoids and polyketides	6
Organosulfur compounds	5
Alkaloids and derivatives	4
Organophosphorus compounds	3

Table S7 Compounds used in validation and application study. P- denotes pesticide, M- denotes mycotoxin and T -transformation compound.

#	CAS	Name	SMILES
M1	88337-96-6	deoxynivalenol-15-acetate	CC1=C[C@@H]2[C@]([C@@H](C1=O)O)([C@]3(C[C@H]([C@H]([C@@]34CO4)O2)O)C)COC(=O)C
P2	30614-22-3	pirimicarb-desmethyl	CC1=C(N=C(N=C1OC(=O)N(C)C)NC)C
P3	100784-20-1	halosulfuron-methyl	CN1C(=C(C(=N1)Cl)C(=O)OC)S(=O)(=O)NC(=O)NC2=NC(=CC(=N2)OC)OC
P4	2310-17-0	phosalone	CCOP(=S)(OCC)SCN1C2=C(C=C(C=C2)Cl)OC1=O
P5	950-35-6	paraoxon-methyl	COP(=O)(OC)OC1=CC=C(C=C1)[N+](=O)[O-]
P6	107-49-3	TEPP	CCOP(=O)(OCC)OP(=O)(OCC)OCC
P7	31972-43-7	fenamiphos-sulfoxide	CCOP(=O)(NC(C)C)OC1=CC(=C(C=C1)S(=O)C)C
P8	3761-41-9	fenthion-sulfoxide	CC1=C(C=CC(=C1)OP(=S)(OC)OC)S(=O)C
P9	72490-01-8	fenoxycarb	CCOC(=O)NCCOC1=CC=C(C=C1)OC2=CC=CC=C2
P10	57018-04-9	tolclofos-methyl	CC1=CC(=C(C(=C1)Cl)OP(=S)(OC)OC)Cl
P11	88671-89-0	myclobutanil	CCCCC(CN1C=NC=N1)(C#N)C2=CC=C(C=C2)Cl
P12	67129-08-2	metazachlor	CC1=C(C(=CC=C1)C)N(CN2C=CC=N2)C(=O)CCl
P13	61213-25-0	flurochloridone	C1C(C(C(=O)N1C2=CC=CC(=C2)C(F)(F)F)Cl)CCl
P14	42874-03-3	oxyfluorfen	CCOC1=C(C=CC(=C1)OC2=C(C=C(C=C2)C(F)(F)F)Cl)[N+](=O)[O-]
P15	34256-82-1	acetochlor	CCC1=CC=CC(=C1N(COCC)C(=O)CCl)C
P16	63284-71-9	nuarimol	C1=CC=C(C(=C1)C(C2=CC=C(C=C2)F)(C3=CN=CN=C3)O)Cl
P17	79983-71-4	hexaconazole	CCCCC(CN1C=NC=N1)(C2=C(C=C(C=C2)Cl)Cl)O
M18	23452-05-3	alternariol-monomethyl ether	CC1=CC(=CC2=C1C3=CC(=CC(=C3C(=O)O2)O)OC)O
P19	55512-33-9	pyridate	CCCCCCCCSC(=O)OC1=CC(=NN=C1C2=CC=CC=C2)Cl
P20	86-86-2	1-naphthylacetamide	C1=CC=C2C(=C1)C=CC=C2CC(=O)N
P21	175013-18-0	pyraclostrobin	COC(=O)N(C1=CC=CC=C1COC2=NN(C=C2)C3=CC=C(C=C3)Cl)OC
M22	1165-39-5	aflatoxin G1	COC1=C2C3=C(C(=O)OCC3)C(=O)OC2=C4C5C=COC5OC4=C1
P23	74115-24-5	clofentezine	C1=CC=C(C(=C1)C2=NN=C(N=N2)C3=CC=CC=C3Cl)Cl
P24	112281-77-3	tetraconazole	C1=CC(=C(C=C1Cl)Cl)C(CN2C=NC=N2)COC(C(F)F)(F)F
P25	81777-89-1	clomazone	CC1(CON(C1=O)CC2=CC=CC=C2Cl)C
P26	24579-73-5	propamocarb	CCCOC(=O)NCCCN(C)C

#	CAS	Name	SMILES
P27	119168-77-3	tebufenpyrad	CCC1=NN(C(=C1Cl)C(=O)NCC2=CC=C(C=C2)C(C)(C)C)C
P28	135410-20-7	acetamiprid	CC(=NC#N)N(C)CC1=CN=C(C=C1)Cl
P29	10265-92-6	methamidophos	COP(=O)(N)SC
P30	173584-44-6	indoxacarb	COC(=O)[C@]12CC3=C(C1=NN(CO2)C(=O)N(C4=CC=C(C=C4)OC(F)(F)F)C(=O)OC)C=CC(=C3)Cl
P31	77732-09-3	oxadixyl	CC1=C(C(=CC=C1)C)N(C(=O)COC)N2CCOC2=O
P32	119-12-0	pyridaphenthion	CCOP(=S)(OCC)OC1=NN(C(=O)C=C1)C2=CC=CC=C2
P33	470-90-6	chlorfenvinphos	CCOP(=O)(OCC)O/C(=C\Cl)/C1=C(C=C(C=C1)Cl)Cl
M34	2270-40-8	diacetoxyscirpenol	CC1=C[C@@H]2[C@](CC1)([C@]3([C@@H]([C@H]([C@H]([C@]34CO4)O2)O)OC(=O)C)C)COC(=O)C
M35	1162-65-8	aflatoxin B1	COC1=C2C3=C(C(=O)CC3)C(=O)OC2=C4[C@@H]5C=CO[C@@H]5OC4=C1
T1	620-08-6	4-methoxypyridine	COc1ccncc1
T2	271-44-3	indazole	[nH]1ncc2ccccc12
T3	123-08-0	4-hydroxybenzaldehyde	Oc1ccc(C=O)cc1
T4	66-40-0	tetraethylammonium	CC[N+](CC)(CC)CC
T5	123-11-5	*p*-anisaldehyde	COc1ccc(C=O)cc1
T6	934-00-9	3-methoxycatechol	COc1cccc(O)c1O
T7	102-69-2	tripropylamine	CCCN(CCC)CCC
T8	56-85-9	glutamine	N[C@@H](CCC(N)=O)C(O)=O
T9	140-10-3	cinnamic acid	OC(=O)C=Cc1ccccc1
T10	15799-79-8	3-methoxy-*N,N*-dimethylaniline	COc1cccc(c1)N(C)C
T11	100-22-1	4-dimethylamino-*N,N*-dimethylaniline	CN(C)c1ccc(cc1)N(C)C
T12	88-99-3	phthalic acid	OC(=O)c1ccccc1C(O)=O
T13	66-71-7	1,10-phenanthroline	c1cnc2c(c1)ccc3cccnc23
T14	13010-31-6	tetrapropylammonium	CCC[N+](CCC)(CCC)CCC
T15	140-40-9	2-acetamido-5-nitrothiazole	CC(=O)Nc1sc(cn1)[N+]([O-])=O
T16	41394-05-2	metamitrone	CC1=NN=C(c2ccccc2)C(=O)N1N

#	CAS	Name	SMILES
T17	34123-59-6	isoproturon	CC(C)c1ccc(NC(=O)N(C)C)cc1
T18	1646-88-4	aldicarb-sulfone	CNC(=O)O\N=C\C(C)(C)[S](C)(=O)=O
T19	15972-60-8	alachlor	CCc1cccc(CC)c1N(COC)C(=O)CCl
T20	13067-93-1	cyanophenphos	CCO[P](=S)(Oc1ccc(cc1)C#N)c2ccccc2
T21	51-34-3	scopolamine	CN1[C@@H]2CC(C[C@H]1[C@H]3[C@@H]2O3)OC(=O)[C@H](CO)C4=CC=CC=C4
T22	2597-03-7	phenthoate	CCOC(=O)C(S[P](=S)(OC)OC)c1ccccc1
T23	23564-05-8	thiophanate-methyl	COC(=O)NC(=S)NC1=CC=CC=C1NC(=S)NC(=O)OC
T24	112281-77-3	tetraconazole	FC(F)C(F)(F)OCC(Cn1cncn1)c2ccc(Cl)cc2Cl
T25	417706-59-3	4-CF_3-C_6H_4-P(pyrr)	FC(F)(F)c1ccc(N=P(N2CCCC2)(N2CCCC2)N2CCCC2)cc1
T26	2097489-41-1	4-[2-(4-nitrophenyl)diazenyl]-*N*-(phenyldi-1-pyrrolidinylphosphoranylidene)benzenamine	O=[N+]([O-])c1ccc(N=Nc2ccc(N=P(c3ccccc3)(N3CCCC3)N3CCCC3)cc2)cc1
T27	2097489-43-3	4-[2-[4-[(diphenyl-1-pyrrolidinylphosphoranylidene)amino]phenyl]diazenyl]-*N,N*-dimethyl-benzenamine	CN(C)c1ccc(N=Nc2ccc(N=P(c3ccccc3)(c3ccccc3)N3CCCC3)cc2)cc1
T28	2097489-43-3	*N,N*-dimethyl-4-[2-[4-[(triphenylphosphoranylidene)amino]phenyl]diazenyl]-benzenamine	CN(C)c1ccc(N=Nc2ccc(N=P(c3ccccc3)(c3ccccc3)N3CCCC3)cc2)cc1
T29	33354-65-3	2-Cl-C_6H_4-P_2(pyrr)	CN(C)c1ccc(N=Nc2ccc(N=P(c3ccccc3)(c3ccccc3)c3ccccc3)cc2)cc1
T30	417706-58-2	Phe-Phe-Phe-Phe	Clc1ccccc1N=P(N=P(N1CCCC1)(N1CCCC1)N1CCCC1)(N1CCCC1)N1CCCC1
T31	2667-02-9	reserpine	NC(Cc1ccccc1)C(=O)NC(Cc1ccccc1)C(=O)NC(Cc1ccccc1)C(=O)NC(Cc1ccccc1)C(=O)O

Table S8 Instruments used to study ionization efficiencies in this study.

Usage	Source	Mass analyzer	Name	Vendor	Lab
Ionization efficiency model development	ESI	Ion trap	Agilent XCT	Agilent	University of Tartu
Ionization efficiency model development	ESI	Linear ion trap	Thermo LTQ	Thermo Fisher Scientific	Janssen Pharmaceutica
Ionization efficiency model development	HESI–II	Linear ion trap	Thermo LTQ	Thermo Fisher Scientific	Janssen Pharmaceutica
Ionization efficiency model development	Z-spray	Quadrupole time-of-flight	Waters Synapt G.2	Waters	Janssen Pharmaceutica
Ionization efficiency model development	JetStream	Single quadrupole	Agilent Single Quad 6100	Agilent	University Lyon 1
Ionization efficiency model development	ESI	Triple quadrupole	Agilent 6495	Agilent	University of Tartu
Ionization efficiency model development/ Validation	JetStream	Triple quadrupole	Agilent 6495	Agilent	University of Tartu
Ionization efficiency model development	TurboSpray	Triple quadrupole	Sciex API 4000	Sciex	Janssen Pharmaceutica
Ionization efficiency model development	ESI	Triple quadrupole	Varian J-320	Varian	University of Tartu

Table S9 Significant descriptors in ESI positive mode model.

Descriptor type	*N*	Descriptor names
Acidic group count	1	nAcid
AlogP[49]	2	ALogP, ALogp2
Atom count	5	nC, nH, nHeavyAtom, nN, nX
Autocorrelation[50]	208	AATS0i, AATS0p, AATS1e, AATS1i, AATS1p, AATS1s, AATS1v, AATS2i, AATS2p, AATS2s, AATS3e, AATS3i, AATS3m, AATS3s, AATS4e, AATS4i, AATS4m, AATS4p, AATS4s, AATS5i, AATS5s, AATS5v, AATS6i, AATS6p, AATS6s, AATS7i, AATS7m, AATS8e, AATS8i, AATS8m, AATS8p, AATS8v, AATSC0e, AATSC0i, AATSC0p, AATSC1e, AATSC1i, AATSC1m, AATSC1p, AATSC1s, AATSC1v, AATSC2e, AATSC2i, AATSC2m, AATSC2s, AATSC2v, AATSC3c, AATSC3e, AATSC3i, AATSC3s, AATSC3v, AATSC4c, AATSC4e, AATSC4i, AATSC4m, AATSC4s, AATSC4v, AATSC5i, AATSC5m, ATSC5v, AATSC6e, AATSC6i, AATSC6s, AATSC6v, AATSC7e, AATSC7i, AATSC7m, AATSC7v, AATSC8e, AATSC8i, AATSC8m, AATSC8p, AATSC8s, AATSC8v, ATS0e, ATS0i, ATS0m, ATS0s, ATS0v, ATS1i, ATS1s, ATS2e, ATS2s, ATS2v, ATS3i, ATS3s, ATS4i, ATS4p, ATS5m, ATS8e, ATS8i, ATS8s, ATSC0i, ATSC0m, ATSC0p, ATSC1c, ATSC1e, ATSC1m, ATSC1p, ATSC1s, ATSC2c, ATSC2e, ATSC2i, ATSC2m, ATSC2s, ATSC3c, ATSC3e, ATSC3m, ATSC3p, ATSC3v, ATSC4c, ATSC4e, ATSC4i, ATSC4m, ATSC4p, ATSC4s, ATSC4v, ATSC5c, ATSC5i, ATSC5m, ATSC5p, ATSC5s, ATSC6c, ATSC6e, ATSC6m, ATSC6p, ATSC6s, ATSC6v, ATSC7i, ATSC7m, ATSC8c, ATSC8e, ATSC8i, ATSC8s, ATSC8v, GATS1c, GATS1e, GATS1i, GATS1m, GATS1p, GATS1s, GATS2c, GATS2e, GATS2i, GATS2m, GATS2p, GATS2s, GATS2v, GATS3c, GATS3m, GATS3p, GATS3s, GATS3v, GATS4c, GATS4e, GATS4m, GATS4p, GATS4s, GATS4v, GATS5c, GATS5e, GATS5i, GATS5m, GATS5v, GATS6e, GATS6p, GATS6s, GATS6v, GATS7m, GATS8c, GATS8e, GATS8i, GATS8p, GATS8v, MATS1i, MATS1p, MATS1s, MATS2c, MATS2e, MATS2m, MATS2v, MATS3c, MATS3e, MATS3i, MATS3m, MATS3s, MATS3v, MATS4e, MATS4i, MATS4m, MATS4p, MATS4s, MATS4v, MATS5c, MATS5e, MATS5m, MATS5p, MATS5s, MATS5v, MATS6e, MATS6p, MATS6s, MATS7c, MATS8c, MATS8i, MATS8m, MATS8s, MATS8v
Barysz matrix[50]	41	EE_Dzi, EE_Dzm, EE_Dzp, EE_Dzs, EE_DzZ, SM1_Dzi, SM1_Dzp, SM1_Dzs, SM1_DzZ, SM1_Dzv, SpAD_Dzp, SpAD_DzZ, SpMax_Dze, SpMax_Dzi, VE1_Dzi, VE1_Dzm, VE1_Dzp, VE1_Dzs, VE1_DzZ, VE1_Dzv, VE2_Dze, VE2_Dzi, VE2_Dzs, VE2_DzZ, VE2_Dzv, VE3_Dzp, VE3_Dzs, VE3_DzZ, VE3_Dzv, VR1_Dzi, VR1_Dzm, VR1_Dzp, VR1_Dzs, VR1_DzZ, VR1_Dzv, VR2_Dzi, VR2_Dzp, VR2_Dzs, VR2_DzZ, VR2_Dzv, VR3_Dzs
Burden modified eigenvalues[50]	55	SpMax1_Bhs, SpMax2_Bhe, SpMax2_Bhv, SpMax3_Bhe, SpMax3_Bhi, SpMax3_Bhm, SpMax3_Bhp, SpMax4_Bhe, SpMax4_Bhi, SpMax4_Bhs, SpMax5_Bhp, SpMax5_Bhv, SpMax6_Bhe, SpMax6_Bhi,

Descriptor type	*N*	Descriptor names
		SpMax6_Bhp, SpMax6_Bhv, SpMax7_Bhm, SpMax7_Bhp, SpMax7_Bhs, SpMax8_Bhe, SpMax8_Bhm, SpMax8_Bhp, SpMax8_Bhs, SpMin1_Bhi, SpMin1_Bhm, SpMin1_Bhp, SpMin1_Bhs, SpMin1_Bhv, SpMin2_Bhi, SpMin2_Bhp, SpMin2_Bhs, SpMin2_Bhv, SpMin3_Bhe, SpMin3_Bhi, SpMin3_Bhm, SpMin3_Bhs, SpMin3_Bhv, SpMin4_Bhe, SpMin4_Bhi, SpMin4_Bhs, SpMin4_Bhv, SpMin5_Bhe, SpMin5_Bhs, SpMin5_Bhv, SpMin6_Bhe, SpMin6_Bhi, SpMin6_Bhv, SpMin7_Bhm, SpMin7_Bhp, SpMin7_Bhs, SpMin7_Bhv, SpMin8_Bhe, SpMin8_Bhi, SpMin8_Bhp, SpMin8_Bhv
Constitutional	4	Mi, Mse, Mv, Sv
Crippen[51]	1	CrippenLogP
Detour matrix[50]	6	EE_Dt, VE1_Dt, VE2_Dt, VE3_Dt, VR1_Dt, VR2_Dt
Atom type electrotopological state[52]	67	DELS, DELS2, gmax, gmin, hmin, maxaaaC, MAXDN, maxdNH, MAXDP, MAXDP2, maxdsCH, maxdssC, maxHAvin, maxHBint4, maxHBint5, maxHCHnX, maxHdCH2, maxHdsCH, maxHsNH2, maxHsSH, maxsNH3p, maxsOH, maxssPH, maxssssBm, maxtN, maxwHBa, maxwHBd, minaaN, minaaS, mindO, mindsCH, mindsN, mindssC, minHBa, minHBd, minHBint5, minHdsCH, minsAsH2, minsNH2, minsOH, minsSeH, minssNH, minsssCH, minssSe, minsssN, minwHBa, nddssSe, ndO, nHsOH, nsCH3, nsSnH3, nssO, nssSe, SaaCH, SdO, SdS, SdssC, SdsssP, SHBa, SHBint9, SHdsCH, SHsNH2, SsNH2, SsNH3p, SsSnH3, SsssCH, sumI
Eluent	5	NH4, pH_aq, polarity_index, surface_tension, viscosity
Extended topochemical atom[53]	18	ETA_Alpha, ETA_Beta, ETA_Beta_ns, ETA_Beta_s, ETA_BetaP, ETA_dAlpha_B, ETA_dBeta, ETA_dBetaP, ETA_dEpsilon_A, ETA_Epsilon_5, ETA_Eta_B, ETA_Eta_B_RC, ETA_Eta_F_L, ETA_Eta_L, ETA_EtaP_B, ETA_EtaP_B_RC, ETA_EtaP_F_L, ETA_Shape_Y
Hybridization ratio	1	HybRatio
Information content[50]	24	BIC0, BIC1, BIC3, BIC4, BIC5, CIC0, CIC1, CIC3, CIC4, CIC5, IC1, IC2, IC3, IC4, MIC0, MIC4, SIC3, SIC4, SIC5, ZMIC1, ZMIC2, ZMIC4, TIC1, TIC5
Molecular distance edge[54]	7	MDEC-11, MDEC-14, MDEC-24, MDEC-34, MDEN-13, MDEN-22, MDEN-33
Molecular linear free energy relation[55]	3	MLFER_BH, MLFER_E, MLFER_S
Bond count	1	nBondsM
Carbon types	1	C2SP1

Table S10 Significant descriptors in ESI negative mode model.

Descriptor type	*N*	Descriptor names
Atom count	1	nX
Autocorrelation[50]	57	AATS1e, AATS1i, AATS2p, AATS2v, AATS3e, AATS4e, AATS5p, AATS8s, AATSC0c, AATSC1p, AATSC3c, AATSC3p, AATSC3v, AATSC4c, AATSC5p, AATSC6v, AATSC7p, ATS0m, ATS0s, ATS1m, ATS1s, ATS3i, ATS3m, ATS3s, ATS4m, ATSC0s, ATSC0v, ATSC1v, ATSC3c, ATSC3p, ATSC4e, ATSC8v, GATS1e, GATS1v, GATS2e, GATS3c, GATS3i, GATS4m, GATS4v, GATS5i, GATS5m, GATS5s, GATS6v, GATS8c, GATS8m, GATS8p, MATS1m, MATS1p, MATS2e, MATS2s, MATS2v, MATS3i, MATS3m, MATS3v, MATS6m, MATS8i, MATS8s
Barysz matrix[50]	32	EE_Dzm, EE_DzZ, SM1_Dze, SM1_Dzi, SM1_Dzm, SM1_Dzs, SM1_Dzv, SpAD_Dzp, SpAD_Dzv, SpDiam_Dze, SpMAD_DzZ, VE1_Dzi, VE1_Dzp, VE1_DzZ, VE2_Dze, VE2_Dzi, VE2_Dzp, VE2_DzZ, VE2_Dzv, VE3_Dze, VE3_Dzi, VE3_Dzm, VE3_Dzp, VE3_Dzs, VE3_DzZ, VR1_Dze, VR1_Dzi, VR1_Dzp, VR1_DzZ, VR1_Dzv, VR3_Dzm, VR3_Dzs
BCUT[56]	1	BCUTc-1l
Burden modified eigenvalue[50]	19	SpMax1_Bhm, SpMax1_Bhp, SpMax2_Bhm, SpMax2_Bhp, SpMax2_Bhs, SpMax3_Bhs, SpMax7_Bhe, SpMax7_Bhi, SpMax8_Bhp, SpMin1_Bhe, SpMin1_Bhi, SpMin3_Bhp, SpMin3_Bhs, SpMin4_Bhs, SpMin4_Bhv, SpMin5_Bhe, SpMin5_Bhi, SpMin6_Bhm, SpMin7_Bhm
Chi Chain[57]	1	VCH-6
Detour matrix[50]	1	VE3_Dt
Atom type electrotopological state[52]	7	DELS2, maxdsCH, minHCsats, nHBint4, SddssS, SdsCH, SHBd
Eluent	4	pH_aq, polarity_index, surface_tension, viscosity
Extended topochemical atom[53]	6	ETA_BetaP, ETA_dBeta, ETA_Eta_L, ETA_Eta_R, ETA_EtaP_F_L, ETA_Shape_P
Information content[50]	8	BIC2, BIC3, BIC4, BIC5, CIC4, MIC3, SIC1, ZMIC3
Molecular linear free energy relation[55]	1	MLFER_A
Chi path[57]	1	AVP-0
Weighted path[58]	1	WTPT-2
Topological charge[1]	2	GGI3, GGI4
Topological distance matrix[50]	2	VE1_D, VE2_D
Wiener numbers[59]	1	WPATH

Table S11 Ionization efficiency prediction model characteristics: absolute error distribution in log*IE* units and times differences.

	positive model				negative model			
	training set		test set		training set		test set	
	log*IE* units	times difference	log*IE* units	times difference	log*IE* units	times difference	log*IE* units	times difference
RMSE	0.29	1.94	0.48	3.01	0.29	1.97	0.35	2.25
**min**	1.83E-05	1.00	5.61E-04	1.00	1.93E-04	1.00	7.71E-03	1.02
**Q1**	0.07	1.17	0.12	1.31	0.07	1.19	0.10	1.27
**median (Q2)**	0.16	1.43	0.24	1.76	0.16	1.43	0.24	1.74
**average**	0.21	1.61	0.34	2.18	0.21	1.61	0.28	1.90
**Q3**	0.29	1.93	0.45	2.81	0.27	1.87	0.39	2.43
**max**	1.97	92.94	2.51	323.67	2.15	139.89	1.05	11.10

Table S12 TOP 15 most important features in developed models in ESI positive and negative mode.

#	Descriptor names	
	**ESI+**	**ESI−**
1	CIC0	SM1_Dzm
2	ATS4i	GGI4
3	nH	DELS2
4	ATS3i	pH_aq
5	pH_aq	ETA_Eta_R
6	ATS4p	SM1_Dze
7	hmin	ATS1m
8	nN	ATS4m
9	viscosity	ATS0m
10	AATS2s	viscosity
11	ATS1i	surface_tension
12	SpMin4_Bhs	ATS0s
13	NH4	MIC3
14	SpMin5_Bhs	polarity_index
15	surface_tension	ATSC0s

Table S13 Absolute concentration prediction error characteristics based on the validation set.

	times difference
min	1.00
Q1 (25%)	1.38
median (Q2)	2.06
average	5.38
Q3 (50%)	4.41
max	93.55

Table S14 Comparison of errors in concentration prediction by compound in case of pesticides and mycotoxins in cereal matrices using three different approaches.

compound	retention time	mean error (using predicted ionization efficiency)	mean error (using 6 compounds[1] as internal standards and for quantifying using the response factor of the retention time wise nearest internal standard)	mean error (assuming equal response factors and using acetamiprid as internal standard)
methamidophos	0.85	1.75	12.60	7.34
propamocarb	1.23	7.34	2.80	4.80
pirimicarb-desmethyl	1.71	14.71	1142.42	5.92
deoxynivalenol-15-acetate	2.48	51.82	29.69	62.79
acetamiprid	2.79	4.08	2.15	1.47
TEPP	3.10	1.28	1.56	1.52
1-naphthylacetamide	3.14	1.67	1.51	2.55
fenamiphos-sulfoxide	3.15	1.26	2.23	1.26
diacetoxyscirpenol	3.37	10.29	5.01	10.37
aflatoxin G1	3.37	1.46	1.48	2.16
oxadixyl	3.39	1.36	1.35	2.26
paraoxon-methyl	3.43	4.29	1.50	2.94
aflatoxin B1	3.58	1.83	232.94	1.65
fenthion-sulfoxide	3.84	2.57	168.63	1.37
metazachlor	4.34	1.57	320.97	1.86
clomazone	4.45	1.81	169.02	1.50
nuarimol	4.51	1.30	309.76	1.82
alternariol-mono	4.74	14.55	7916.96	45.97
myclobutanil	4.88	1.42	160.12	1.20
halosulfuron-methy	4.93	3.06	283.66	1.65
tetraconazole	5.02	1.78	177.02	1.17
pyridaphenthion	5.03	2.15	135.82	1.34
hexaconazole	5.21	1.47	246.25	1.59
acetochlor	5.28	2.50	897.34	5.21
fenoxycarb	5.28	2.16	437.50	2.77
flurochloridone	5.30	1.79	3718.81	21.59
chlorfenrinphos	5.45	6.32	30.49	1.90
pyraclostrobin	5.84	3.60	5.32	4.09
clofenfezine	5.95	1.40	74.42	4.17
tolclofos-methyl	5.96	2.65	323.54	18.13

compound	retention time	mean error (using predicted ionization efficiency)	mean error (using 6 compounds[1] as internal standards and for quantifying using the response factor of the retention time wise nearest internal standard)	mean error (assuming equal response factors and using acetamiprid as internal standard)
phosalone	5.98	3.33	212.02	11.88
indoxacarb	6.05	8.38	14.29	1.32
tebufenpyrad	6.22	1.70	18.62	1.17
oxyfluorfen	6.42	18.86	1285.53	72.02
pyridate	7.49	3.58	49.44	3.03
**Mean error**		5.46	525.51	8.96

[1]6 internal standards and corresponding retention times:

- glutamine 0.46
- scopolamine 1.69
- 3-methoxycatechol 2.02
- Phe-Phe-Phe-Phe 3.58
- *N,N*-dimethyl-4-[2-[4-[(triphenylphosphoranylidene)amino]phenyl]diazenyl]-benzenamine 5.33
- 2-Cl-C_6H_4-P_2(pyrr) 7.01

Table S15 Comparison of errors in concentration prediction by matrix in case of pesticides and mycotoxins in cereal matrices using ionization efficiency prediction.

matrix	average error
oat	6.3
barley	5.7
maize	5.4
rye	5.4
wheat	5.4
rice	4.8
solvent	4.6

Table S16 Properties of pesticides studied in application study.

See chemical_properties_35_pesticides.xlsx

Table S17 Raw data collected in application study in example of pesticides and mycotoxins in cereals.

See cereals_raw_data.csv

Table S18 Summary of data used for model development and concentration prediction.

	Mode	**ESI+**	**ESI–**
**Ionization efficiency model development**	# of unique compounds	353	101
	# of eluent compositions	106	33
	# of instruments	7	3
	# of labs	3	2
	Methods used	direct infusion, flow injection analysis	direct infusion, flow injection analysis
	**Total of log*IE* values**	**3139**	**1286**
**Concentration prediction (validation)**	# of analytes	35	
	type of analytes	pesticides, mycotoxins	
	the lowest concentration	$3.6\cdot10^{-9}$ M	
	the highest concentration	$3.5\cdot10^{-4}$ M	
	measurement type	LC/ESI/MS gradient elution	
	lab	Tartu	
	sample	cereal	
	# of samples	6	
	**Total of datapoints**	**2233**	

Figure S1 Comparison of the chemical space coverage based on logP values. a – ESI positive mode; b – ESI negative mode. The comparison is made with databases of DrugBank,[60] Human Metabolome Database (HMDB),[61] data used in model development, and data pooled from literature (Table S1) HMDB also includes compounds that have not been and cannot be measured with LC/MS.

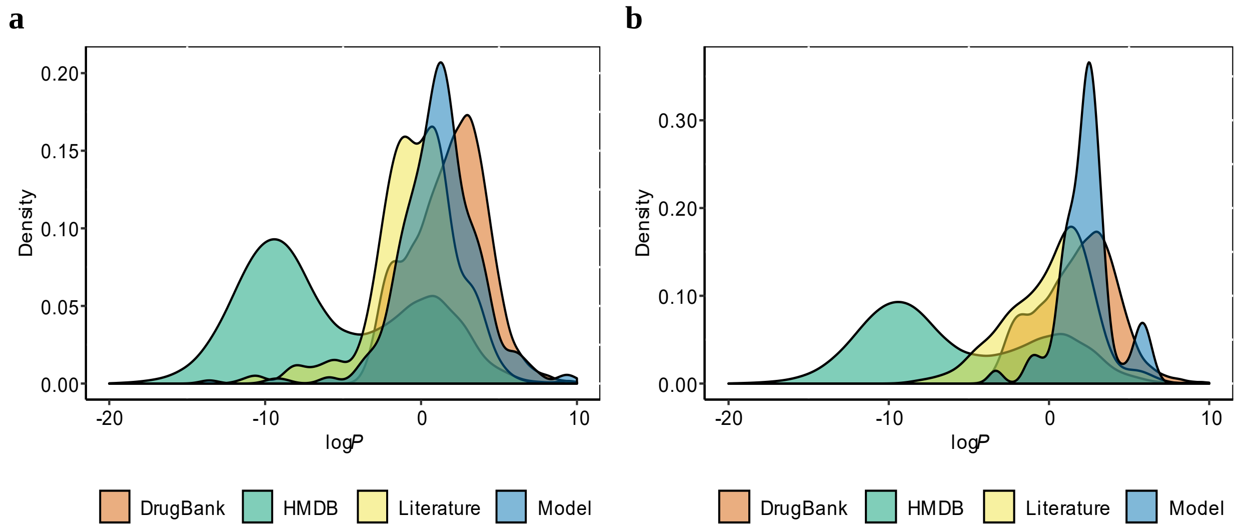

Figure S2 A comparison of the chemical space covered by compounds included in this study (training, test, and validation sets) in comparison to (left) NORMAN, (middle) HMDB, and (right) DrugBank databased. Each dot represents one compound. The compounds from current study are marked with blue and compounds from suspect screening databases are marked with yellow. For NORMAN and HMDB, only the compounds that have LC/MS records available were used. It should be noted that Drugbank also includes proteins and other larger compounds not covered by the proposed method.

The principal component analysis was conducted based on the PaDEL descriptor, and shown are the scores plots from the first two principal components. In all cases first and second principal component explain relatively small part of the total variance (21 to 29%). This is expected, as the chemical space has high dimensionality and includes compounds from very different classes.

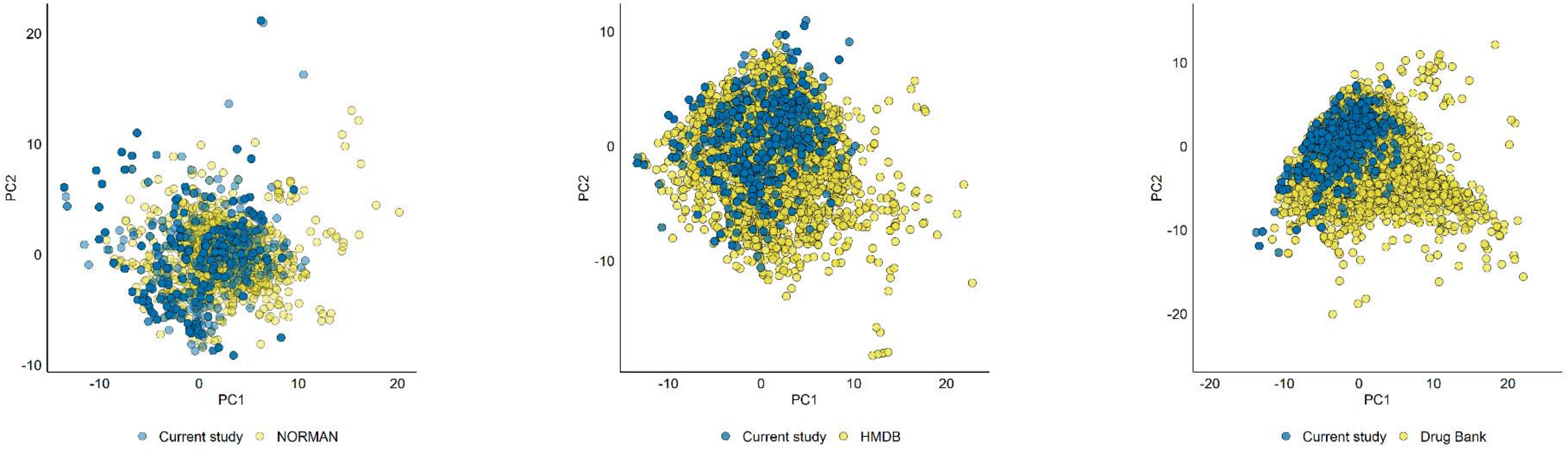

Figure S3 The PCA analysis of the compounds from training, test and validation set based on the PaDEL descriptors. First two principal component explain ca 30% of the total variance. Each dot represents one compound.

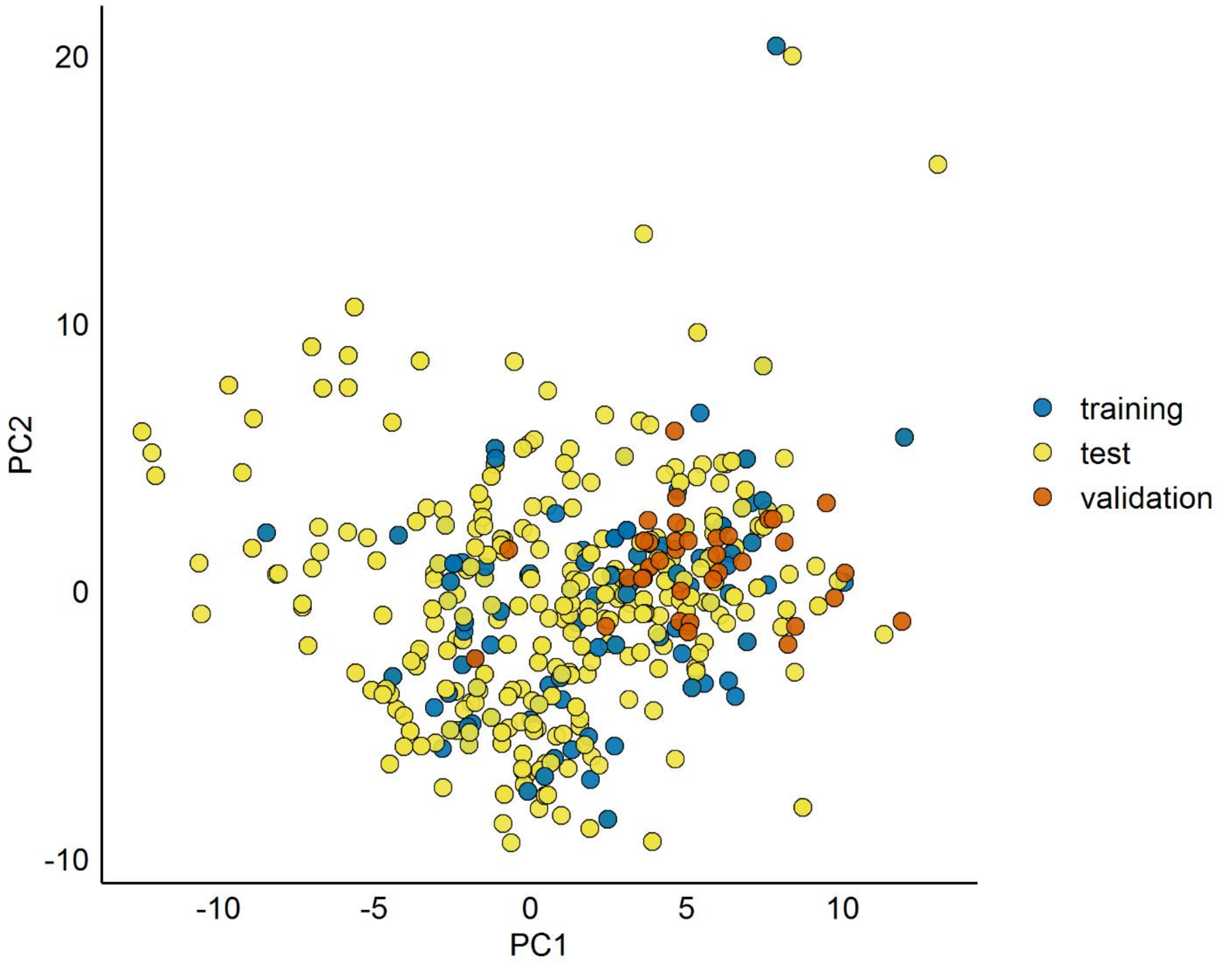

Figure S4 PCA analysis of the training dataset compounds(n = 353). Violet dots denote the compounds used for studying the solvents. For choosing the set to study solvents, 18 first principal components (described variance 70.7%) were used. For clairty the first three principal components are presented. Each dot represesnts one compound.

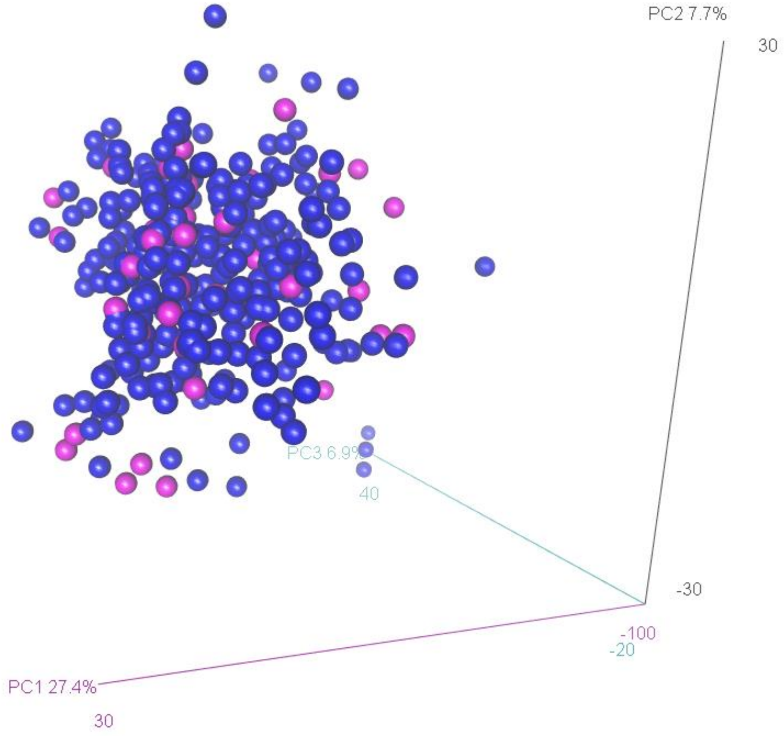

Boxplots

For the boxplots the lower line presents the 1st quartile, the line in the middle the 2nd quartile (median) and the higher line the 3rd quartile. Whiskers are found according to the formula:

$$upper\ whisker = \min(\max(x), Q_3 + 1.5 \cdot IQR) \qquad \text{Eq 5}$$
$$lower\ whisker = \max(\min(x), Q_1 - 1.5 \cdot IQR)$$

where $IQR = Q_3 - Q_1$ and Q_1 represents 1st quartile and Q_3 represents 3rd quartile.

Figure S5 Comparison of prediction errors ionization efficiencies between acetonitrile and methanol containing solvents in ESI positive mode. Results are divided into groups by water phase pH and organic modifier content. Comparison based on intersection of compounds measured in methanol as well as in acetonitrile. The compared results are measured on one instrument. Each datapoint corresponds to one compound solvent combination. Dots represent outliers.

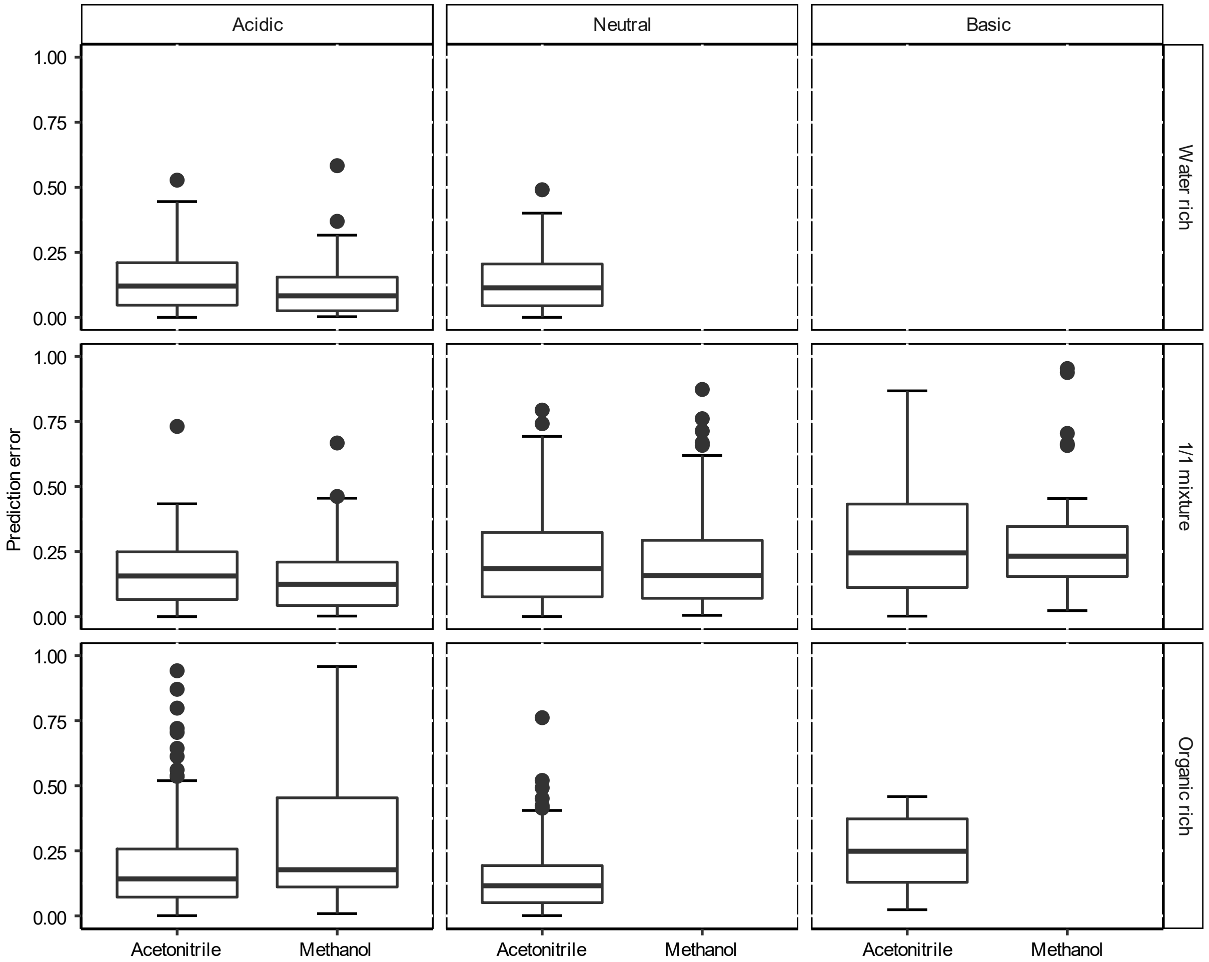

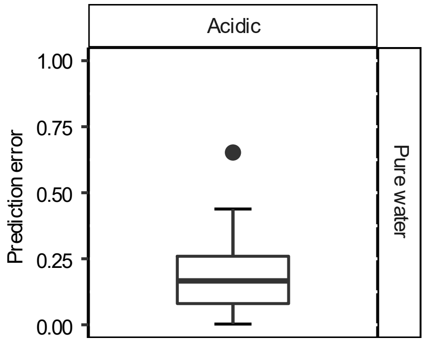

Acidic: pH < 5. **Neutral:** 5 ≤ pH < 8. **Basic:** pH ≥ 8. **Pure water:** organic modifier percentage = 0%. **Water rich:** 0% < organic modifier percentage < 40%. **1/1 mixture:** 40% ≤ organic modifier percentage < 60%. **Organic rich:** 60% ≤ organic modifier percentage < 100%.

Figure S6 Comparison of the prediction error of ionization efficiencies between neat acetonitrile and methanol in ESI positive mode. Divided into groups by pH adjusting additive type. Comparison is based on intersection of compounds measured in methanol as well as in acetonitrile. The compared results are measured on one instrument. Every datapoint corresponds to one compound. Dots represent outliers.

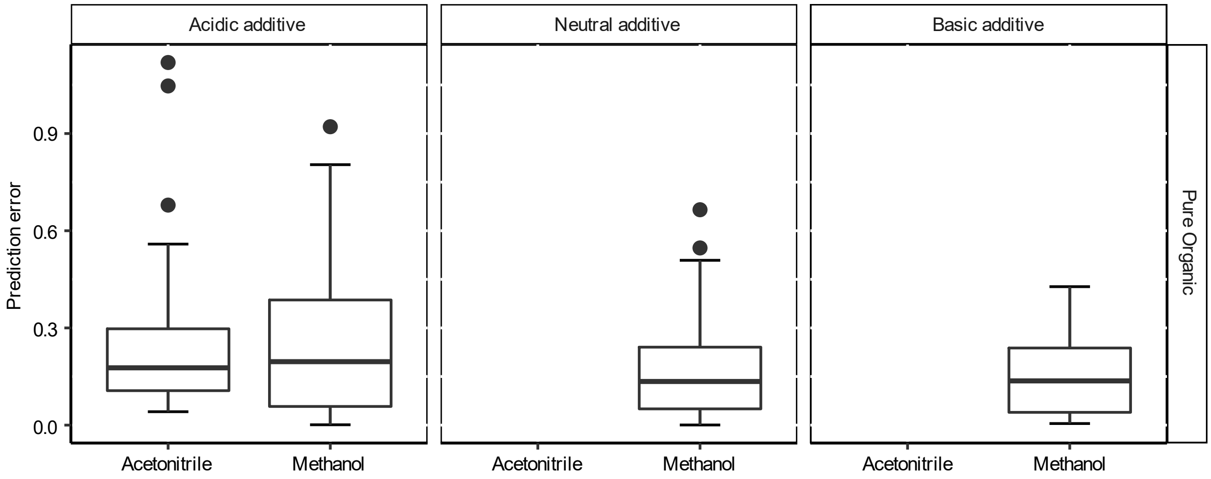

Acidic additive: formic acid, trifluoroacetic acid, oxalic acid, **Neutral additive:** ammonium acetate, ammonium formate, **Basic additive:** ammonia.

Figure S7 Comparison of the prediction error of ionization efficiencies in acetonitrile containing solvents in ESI negative mode. Results are divided into groups by water phase pH and organic modifier content. Comparison based on intersection of compounds measured in all pH groups. The compared results are measured on one instrument. Every datapoint corresponds to one compound. Dots represent outliers.

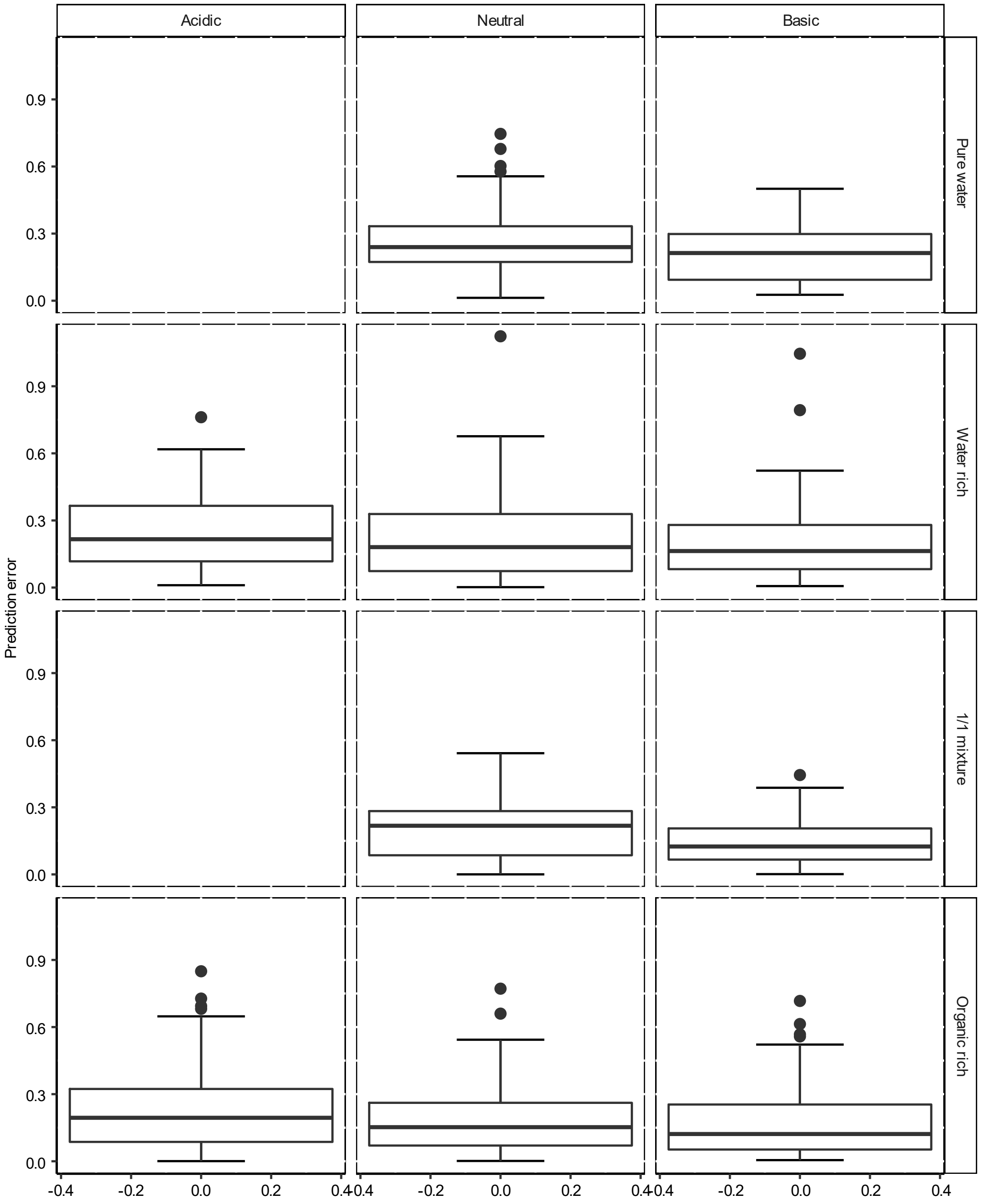

Acidic: pH < 5. **Neutral:** 5 ≤ pH < 8. **Basic:** pH ≥ 8. **Pure water:** organic modifier percentage = 0%. **Water rich:** 0% < organic modifier percentage < 40%. **1/1 mixture:** 40% ≤ organic modifier percentage < 60%. **Organic rich:** 60% ≤ organic modifier percentage < 100%.

Figure S8 Comparison of the prediction error of ionization efficiency between different solvents in ESI negative mode. Compared for the intersection of compounds in studied eluent compositions. Every datapoint corresponds to one compound solvent composition combination. Dots represent outliers.

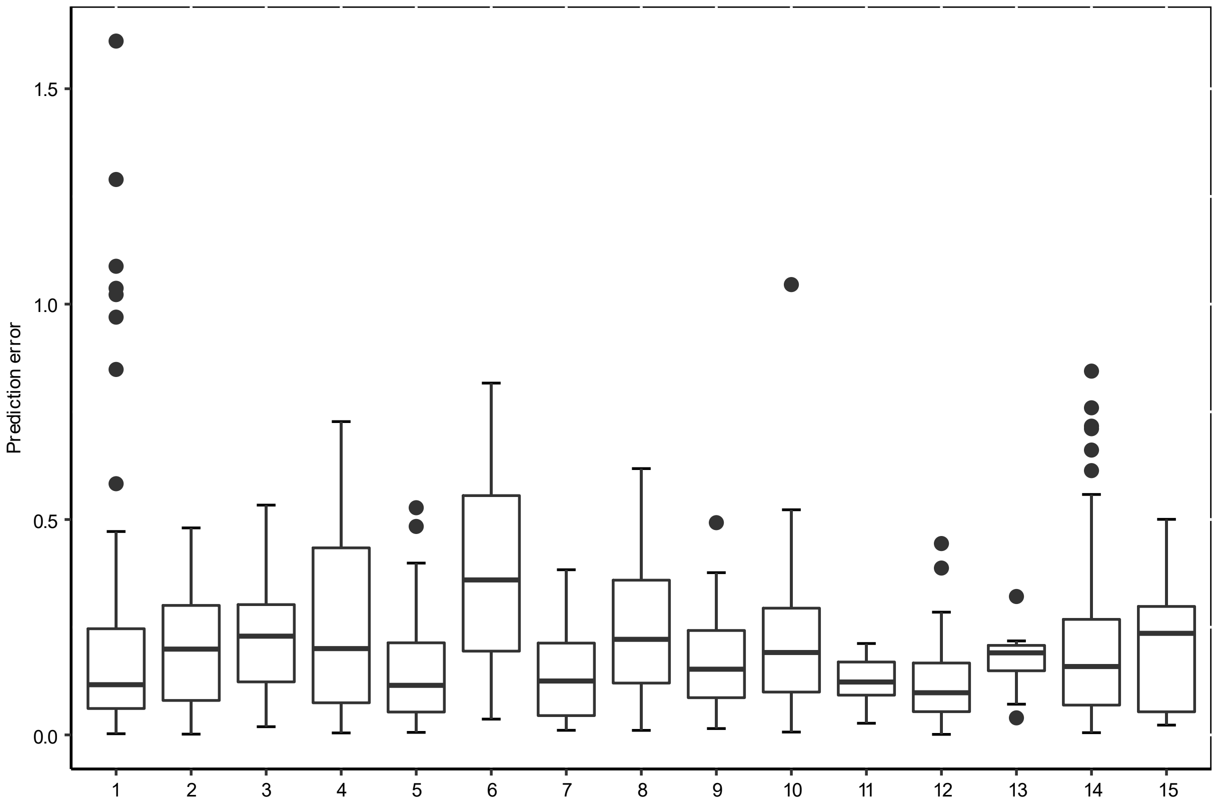

1 - acetonitrile 52 mM ammonia.
2 - acetonitrile/ water phase 20/80 4 mM ammonium acetate pH(aq) = 5.0
3 - acetonitrile/ water phase 80/20 0.2 mM ammonium acetate pH(aq) = 5.0
4 - acetonitrile/ water phase 80/20 1 mM ammonium acetate pH(aq) = 3.45
5 - acetonitrile/ water phase 80/20 1 mM ammonium acetate pH(aq) = 5.0
6 - acetonitrile/ water phase 80/20 1 mM ammonium acetate pH(aq) = 7.0
7 - acetonitrile/ water phase 80/20 1 mM ammonium acetate pH(aq) = 7.8
8 - acetonitrile/ water phase 20/80 21 mM formic acid pH(aq) = 2.78
9 - acetonitrile/ water phase 80/20 5 mM formic acid pH(aq) = 2.78
10 - acetonitrile/ water phase 20/80 41 mM ammonia pH(aq) = 10.5
11 - acetonitrile/ water phase 40/60 31 mM ammonia pH(aq) = 10.5
12 - acetonitrile/ water phase 50/50 26 mM ammonia pH(aq) = 10.5
13 - acetonitrile/ water phase 60/40 21 mM ammonia pH(aq) = 10.5
14 - acetonitrile/ water phase 80/20 10 mM ammonia pH(aq) = 10.5
15 – 52 mM ammonia pH(aq) = 10.5

Figure S9 Correlation between log*IE* values measured for set of compounds in the same eluent composition on different mass spectrometric setups. The intersection of compound-solvent cominations studied with Agilent XCT and Waters Synapt G2 is too few to study the correlations. Each dot represents one compound-solvent pair.

a.

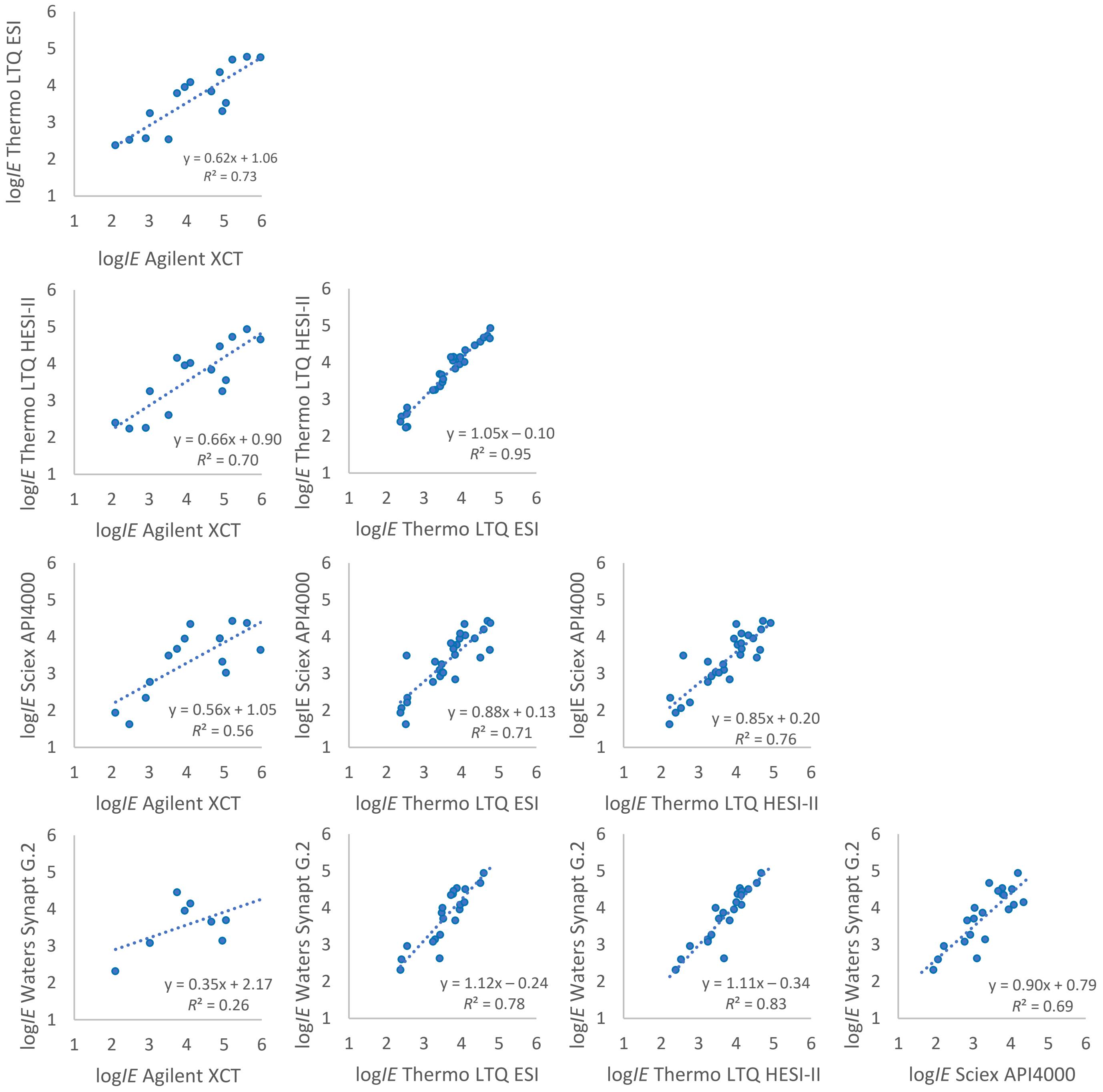

b

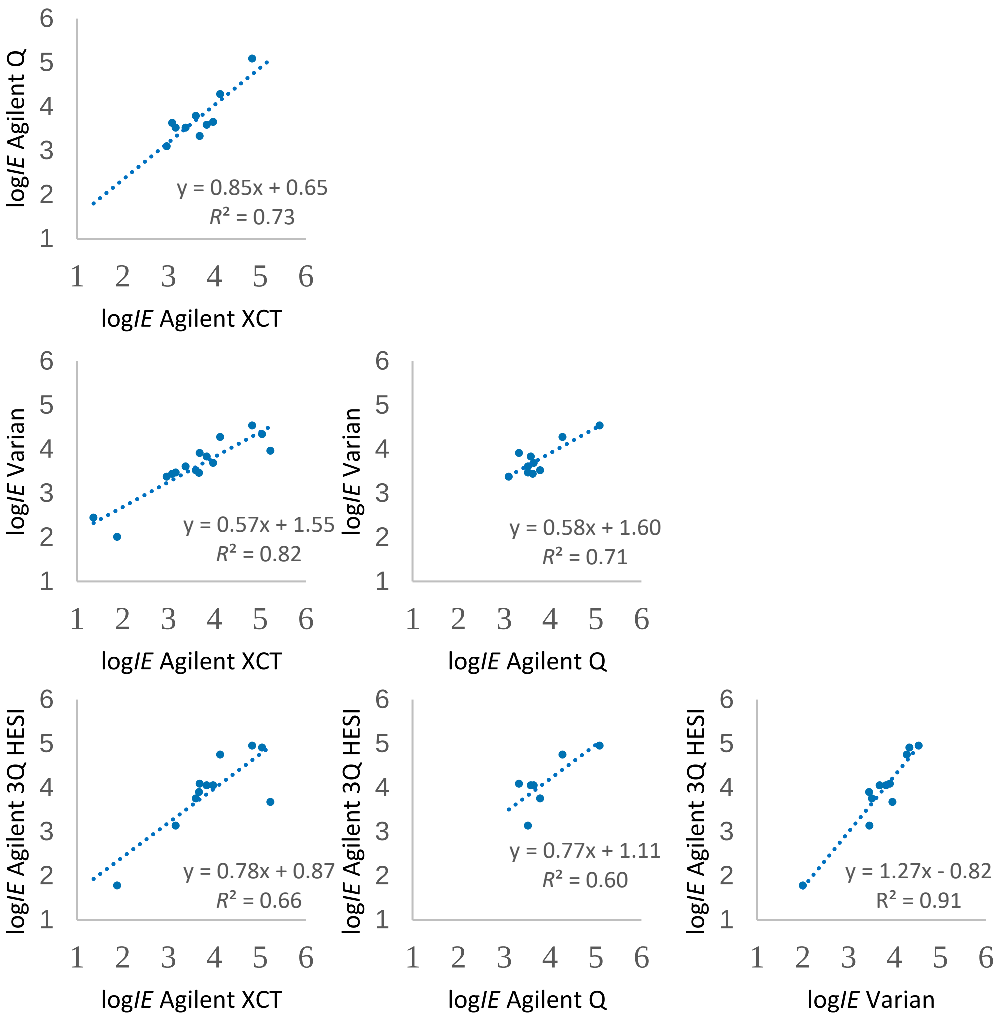
log*IE* Agilent Q
y = 0.85x + 0.65
R^2 = 0.73
log*IE* Agilent XCT
log*IE* Varian
y = 0.57x + 1.55
R^2 = 0.82
log*IE* Agilent XCT
log*IE* Varian
y = 0.58x + 1.60
R^2 = 0.71
log*IE* Agilent Q
log*IE* Agilent 3Q HESI
y = 0.78x + 0.87
R^2 = 0.66
log*IE* Agilent XCT
log*IE* Agilent 3Q HESI
y = 0.77x + 1.11
R^2 = 0.60
log*IE* Agilent Q
log*IE* Agilent 3Q HESI
y = 1.27x - 0.82
R^2 = 0.91
log*IE* Varian

Figure S10 Comparison of the prediction error of ionization efficiency values for different instruments (Table S7) in ESI positive mode. Every datapoint corresponds to one compound solvent composition combination. Dots represent outliers.

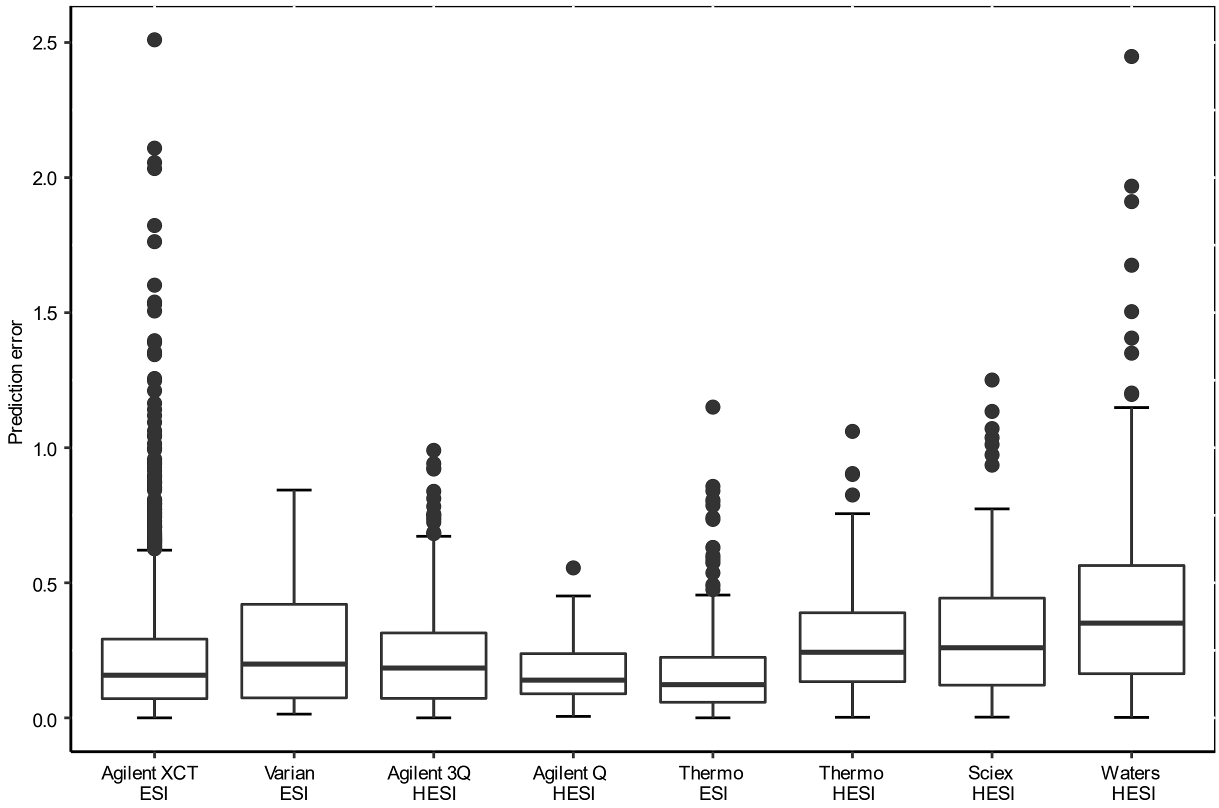

Figure S11 The predicted ionization efficiency values for the validation compounds relative to the measured values. The validation set included 35 pesticides and mycotoxines. Every datapoint corresponds to one compound solvent composition combination. All measurements have been done on Agilent 6495 triple quadrupole instrument with Jet Streem ionization source.

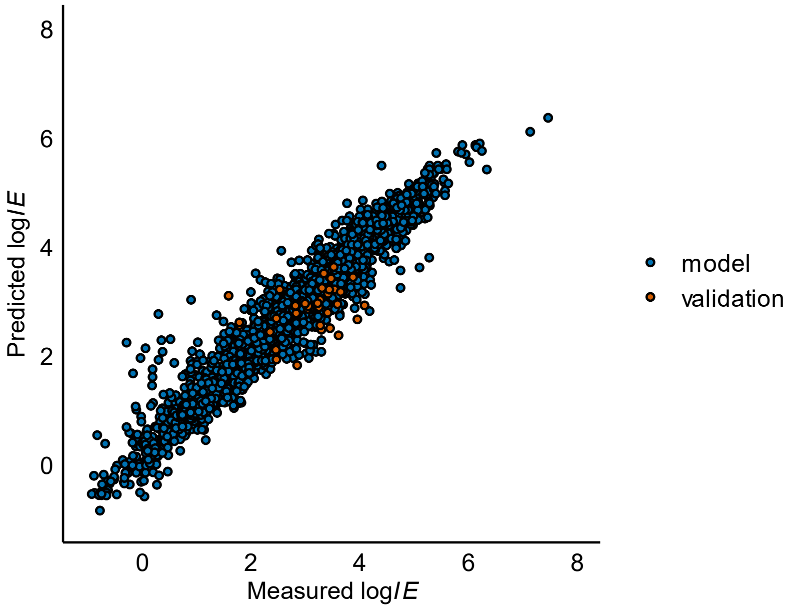

Figure S12 Comparison of predicted and spiked concentration in case of pesticides in cereal samples.

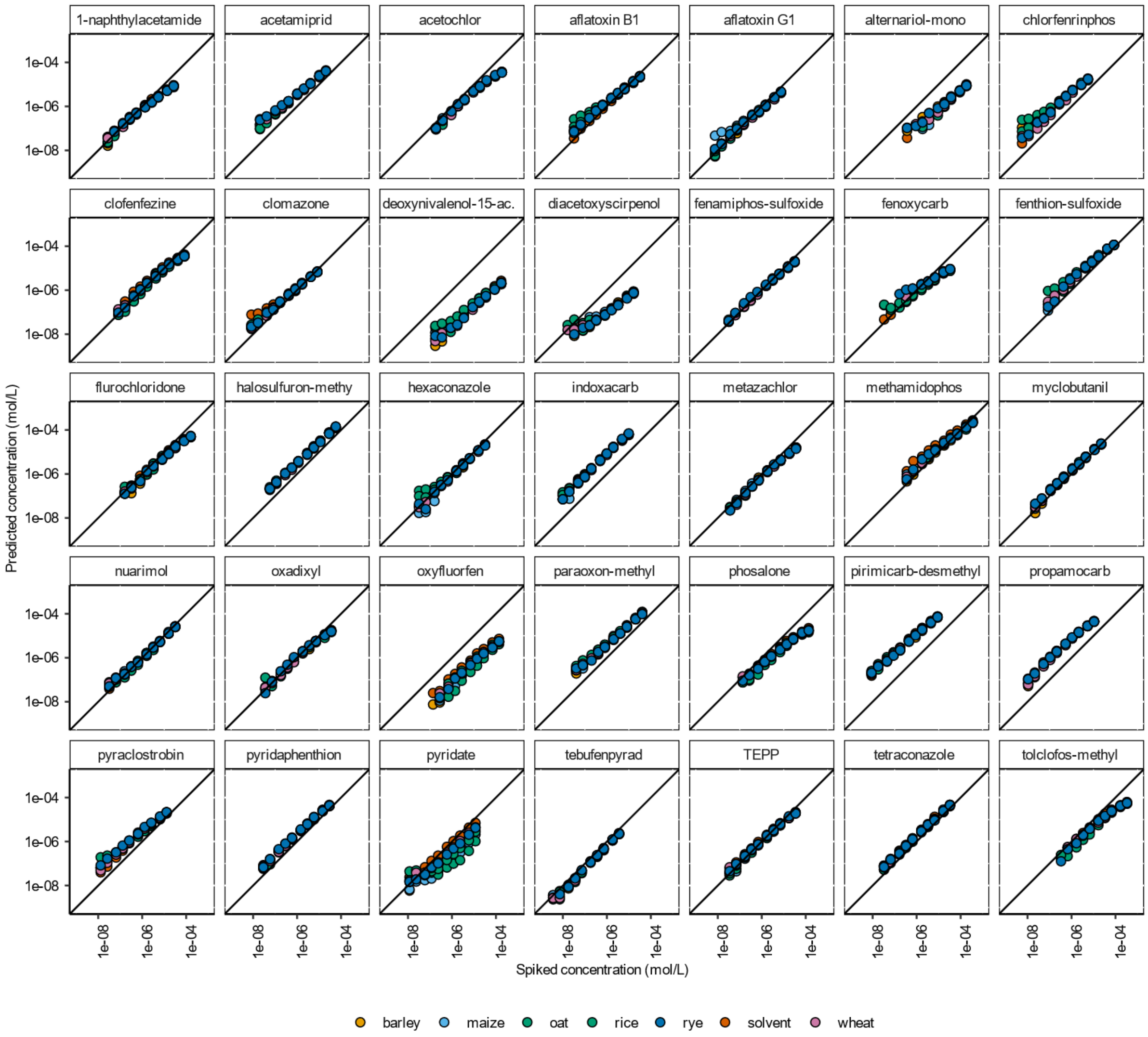

Figure S13 Comparison of the prediction error of ionization efficiencies for different ionization efficiency groups in ESI positive mode. Every datapoint corresponds to one compound-solvent combination. Dots represent outliers.

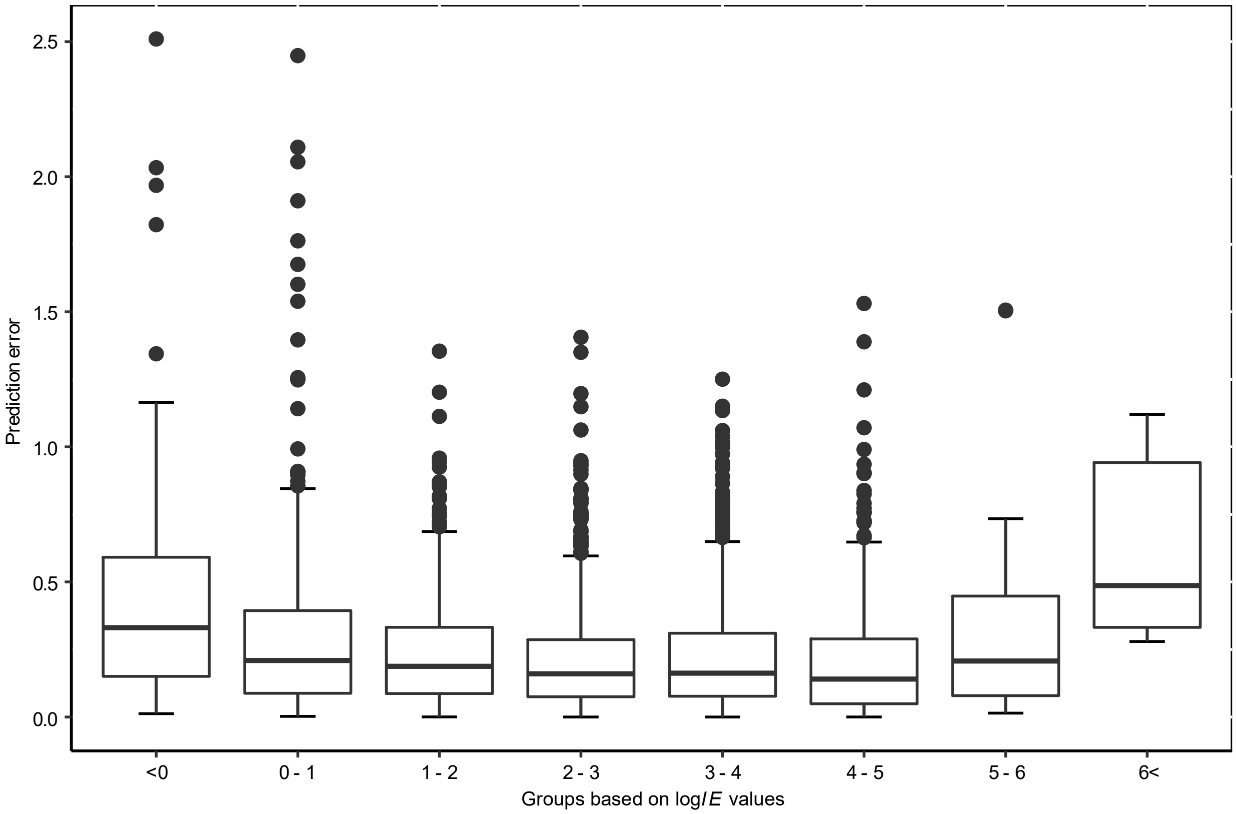

Figure S14 Comparison of the prediction error of ionization efficiencies for different ionization efficiency groups in ESI negative mode. Every datapoint corresponds to one compound-solvent combination. Dots represent outliers.

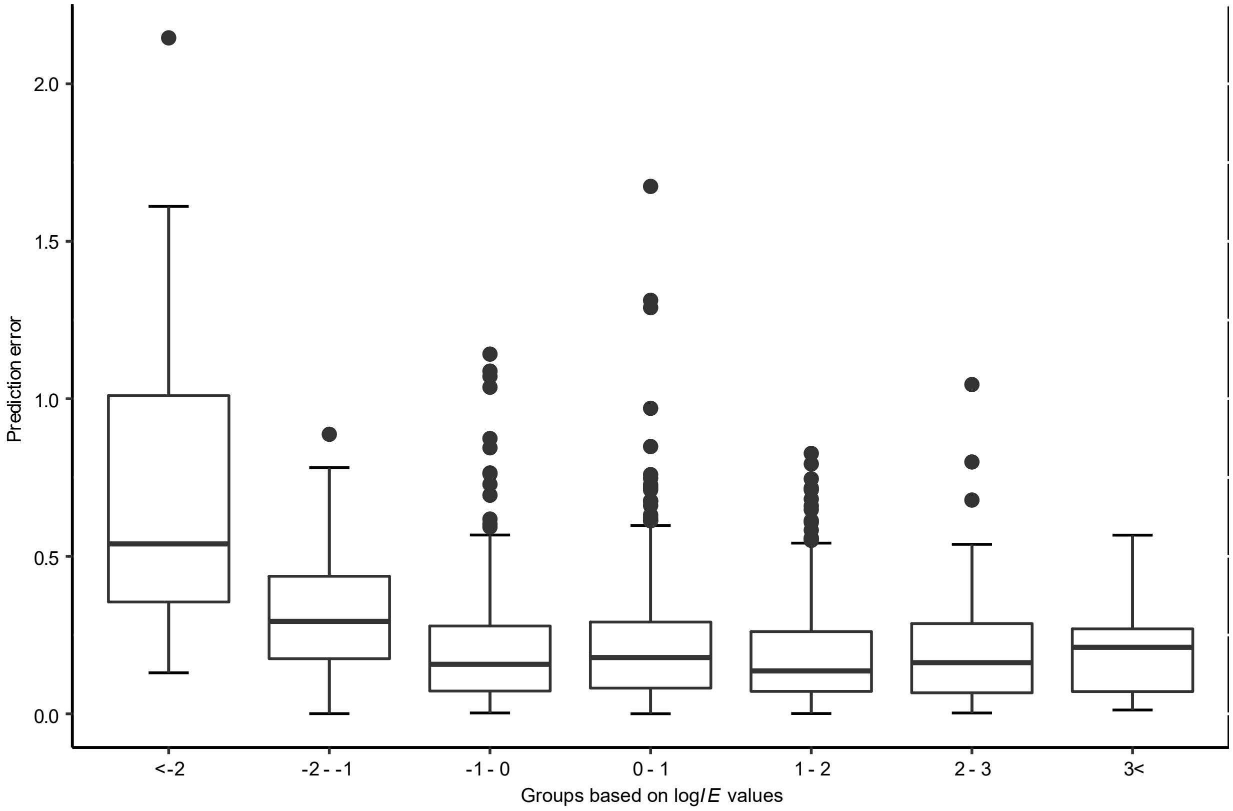

Code S1 Code used for model development

```
library(tidyverse)
library(caTools)
#input data with eluent and compound descriptors
dataset

# Splitting the dataset into the Training set and Test set
set.seed(1000)
split <-  sample.split(dataset %>% select(logIE),
                       SplitRatio = 0.8)
dataset <- dataset %>%
  mutate(SPLIT = split)

training_set <- filter(dataset,
                       SPLIT == TRUE)
test_set <- filter(dataset,
                   SPLIT == FALSE)
#Selecting only parameters that are needed for model development
training_set <- select(training_set, -SPLIT)
test_set <- select(test_set, -SPLIT)

# Random forest regression requires y as vector and features as matrix
y_train <-  as.vector(training_set$logIE)
y_test <-  as.vector(test_set$logIE)
x_train <-  as.matrix(training_set[,-1])
x_test <-  as.matrix(test_set[,-1])

# Regularized random forest regression
regressor <-  RRF(x = x_train,
                  y = y_train,
                  xtest = x_test,
                  ytest = y_test,
                  flagReg = 0, # 1 regularization applied, 0 regularization
not applied
                  ntree = 100, # number of trees
                  keep.forest=TRUE) # needed to save the optimized random
forest
# Predicting the logIE using developd regressor
training_pred <-  predict(regressor,
                          newdata = training_set,
                          predict.all = TRUE)$aggregate
test_pred <- predict(regressor,
                     newdata = test_set,
                     predict.all = TRUE)$aggregate
```

References

1. Snyder, L. R., Kirkland, J. J. & Dolan, J. W. *Introduction to Modern Liquid Chromatography*. (John Wiley & Sons, Inc., 2009).
2. Mikhail, S. Z. & Kimel, W. R. Densities and Viscosities of 1-Propanol-Water Mixtures. *J. Chem. Eng. Data* **8**, 323–328 (1963).
3. Noda, K., Ohashi, M. & Ishida, K. Viscosities and Densities at 298.15 K for Mixtures of Methanol, Acetone, and Water. *J Chem Eng Data* **27**, 326–328 (1982).
4. Rudakov, O. B., Belyaev, D. S., Khorokhordina, E. A. & Podolina, E. A. Surface tension of binary mobile phases for liquid chromatography. *Russ. J. Phys. Chem. A* **81**, 366–369 (2007).
5. Howard, K. S. & McAllister, R. A. Surface tension of acetone-water solutions up to their normal boiling points. *AIChE J.* **3**, 325–329 (1957).
6. Katz, E., Eksteen, R., Schoenmakers, P. & Miller, N. *Handbook of HPLC*. (M. Dekker, 1998).
7. Alfaro, C. M., Uwakweh, A.-O., Todd, D. A., Ehrmann, B. M. & Cech, N. B. Investigations of Analyte-Specific Response Saturation and Dynamic Range Limitations in Atmospheric Pressure Ionization Mass Spectrometry. *Anal. Chem.* **86**, 10639–10645 (2014).
8. Alymatiri, C. M., Kouskoura, M. G. & Markopoulou, C. K. Decoding the signal response of steroids in electrospray ionization mode (ESI-MS). *Anal Methods* **7**, 10433–10444 (2015).
9. Basiri, B., Murph, M. M. & Bartlett, M. G. Assessing the Interplay between the Physicochemical Parameters of Ion-Pairing Reagents and the Analyte Sequence on the Electrospray Desorption Process for Oligonucleotides. *J. Am. Soc. Mass Spectrom.* **28**, 1647–1656 (2017).
10. Beach, D. G. & Gabryelski, W. Linear and Nonlinear Regimes of Electrospray Signal Response in Analysis of Urine by Electrospray Ionization-High Field Asymmetric Waveform Ion Mobility Spectrometry-MS and Implications for Nontarget Quantification. *Anal. Chem.* **85**, 2127–2134 (2013).
11. Bedner, M. & Duewer, D. L. Dynamic Calibration Approach for Determining Catechins and Gallic Acid in Green Tea Using LC–ESI/MS. *Anal. Chem.* **83**, 6169–6176 (2011).
12. Byrdwell, W. C. Quadruple parallel mass spectrometry for analysis of vitamin D and triacylglycerols in a dietary supplement. *J. Chromatogr. A* **1320**, 48–65 (2013).
13. Caetano, S. *et al.* Exploring and modelling the responses of electrospray and atmospheric pressure chemical ionization techniques based on molecular descriptors. *Anal. Chim. Acta* **550**, 92–106 (2005).
14. Cech, N. B. & Enke, C. G. Relating Electrospray Ionization Response to Nonpolar Character of Small Peptides. *Anal. Chem.* **72**, 2717–2723 (2000).
15. Cech, N. B., Krone, J. R. & Enke, C. G. Predicting Electrospray Response from Chromatographic Retention Time. *Anal. Chem.* **73**, 208–213 (2001).
16. Chalcraft, K. R., Lee, R., Mills, C. & Britz-McKibbin, P. Virtual Quantification of Metabolites by Capillary Electrophoresis-Electrospray Ionization-Mass Spectrometry: Predicting Ionization Efficiency Without Chemical Standards. *Anal. Chem.* **81**, 2506–2515 (2009).
17. Cífková, E. *et al.* Nontargeted Quantitation of Lipid Classes Using Hydrophilic Interaction Liquid Chromatography–Electrospray Ionization Mass Spectrometry with Single Internal Standard and Response Factor Approach. *Anal. Chem.* **84**, 10064–10070 (2012).
18. Cramer, C. J., Johnson, J. L. & Kamel, A. M. Prediction of Mass Spectral Response Factors from Predicted Chemometric Data for Druglike Molecules. *J. Am. Soc. Mass Spectrom.* **28**, 278–285 (2017).

19. Dahal, U. P., Jones, J. P., Davis, J. A. & Rock, D. A. Small Molecule Quantification by Liquid Chromatography-Mass Spectrometry for Metabolites of Drugs and Drug Candidates. *Drug Metab. Dispos.* **39**, 2355–2360 (2011).
20. Ehrmann, B. M., Henriksen, T. & Cech, N. B. Relative importance of basicity in the gas phase and in solution for determining selectivity in electrospray ionization mass spectrometry. *J. Am. Soc. Mass Spectrom.* **19**, 719–728 (2008).
21. Espinosa, M. S., Folguera, L., Magallanes, J. F. & Babay, P. A. Exploring analyte response in an ESI-MS system with different chemometric tools. *Chemom. Intell. Lab. Syst.* **146**, 120–127 (2015).
22. Gao, F. *et al.* A Refined Model for Ionization of Small Molecules in Electrospray Mass Spectrometry. *Chem. Lett.* **45**, 955–957 (2016).
23. Ghosh, B. & Jones, A. D. Dependence of negative-mode electrospray ionization response factors on mobile phase composition and molecular structure for newly-authenticated neutral acylsucrose metabolites. *The Analyst* **140**, 6522–6531 (2015).
24. Gioumouxouzis, C. I., Kouskoura, M. G. & Markopoulou, C. K. Negative electrospray ionization mode in mass spectrometry: A new perspective via modeling. *J. Chromatogr. B* **998–999**, 97–105 (2015).
25. Golubović, J., Birkemeyer, C., Protić, A., Otašević, B. & Zečević, M. Structure–response relationship in electrospray ionization-mass spectrometry of sartans by artificial neural networks. *J. Chromatogr. A* **1438**, 123–132 (2016).
26. Hatsis, P., Waters, N. J. & Argikar, U. A. Implications for Metabolite Quantification by Mass Spectrometry in the Absence of Authentic Standards. *Drug Metab. Dispos.* **45**, 492–496 (2017).
27. Henriksen, T., Juhler, R. K., Svensmark, B. & Cech, N. B. The relative influences of acidity and polarity on responsiveness of small organic molecules to analysis with negative ion electrospray ionization mass spectrometry (ESI-MS). *J. Am. Soc. Mass Spectrom.* **16**, 446–455 (2005).
28. Hermans, J., Ongay, S., Markov, V. & Bischoff, R. Physicochemical Parameters Affecting the Electrospray Ionization Efficiency of Amino Acids after Acylation. *Anal. Chem.* **89**, 9159–9166 (2017).
29. Huffman, B. A., Poltash, M. L. & Hughey, C. A. Effect of Polar Protic and Polar Aprotic Solvents on Negative-Ion Electrospray Ionization and Chromatographic Separation of Small Acidic Molecules. *Anal. Chem.* **84**, 9942–9950 (2012).
30. Kalogiouri, N. P., Aalizadeh, R. & Thomaidis, N. S. Investigating the organic and conventional production type of olive oil with target and suspect screening by LC-QTOF-MS, a novel semi-quantification method using chemical similarity and advanced chemometrics. *Anal. Bioanal. Chem.* **409**, 5413–5426 (2017).
31. Kamga, A. W., Behar, F. & Hatcher, P. G. Quantitative Analysis of Long Chain Fatty Acids Present in a Type I Kerogen Using Electrospray Ionization Fourier Transform Ion Cyclotron Resonance Mass Spectrometry: Compared with BF3/MeOH Methylation/GC-FID. *J. Am. Soc. Mass Spectrom.* **25**, 880–890 (2014).
32. Kiontke, A., Oliveira-Birkmeier, A., Opitz, A. & Birkemeyer, C. Electrospray Ionization Efficiency Is Dependent on Different Molecular Descriptors with Respect to Solvent pH and Instrumental Configuration. *PLOS ONE* **11**, e0167502 (2016).
33. Koivusalo, M., Haimi, P., Heikinheimo, L., Kostiainen, R. & Somerharju, P. Quantitative determination of phospholipid compositions by ESI-MS: effects of acyl chain length, unsaturation, and lipid concentration on instrument response. *J. Lipid Res.* **42**, 663–672 (2001).

34. Leitner, A., Emmert, J., Boerner, K. & Lindner, W. Influence of Solvent Additive Composition on Chromatographic Separation and Sodium Adduct Formation of Peptides in HPLC–ESI MS. *Chromatographia* **65**, 649–653 (2007).
35. Mandra, V. J., Kouskoura, M. G. & Markopoulou, C. K. Using the partial least squares method to model the electrospray ionization response produced by small pharmaceutical molecules in positive mode: Modelling positive electrospray ionization response. *Rapid Commun. Mass Spectrom.* **29**, 1661–1675 (2015).
36. Mehta, N. *et al.* Mass Spectrometric Quantification of N-Linked Glycans by Reference to Exogenous Standards. *J. Proteome Res.* **15**, 2969–2980 (2016).
37. Monnin, C., Ramrup, P., Daigle-Young, C. & Vuckovic, D. Improving negative liquid chromatography/electrospray ionization mass spectrometry lipidomic analysis of human plasma using acetic acid as a mobile-phase additive. *Rapid Commun. Mass Spectrom.* **32**, 201–211 (2018).
38. Nguyen, T. B., Nizkorodov, S. A., Laskin, A. & Laskin, J. An approach toward quantification of organic compounds in complex environmental samples using high-resolution electrospray ionization mass spectrometry. *Anal Methods* **5**, 72–80 (2013).
39. Pieke, E. N., Granby, K., Trier, X. & Smedsgaard, J. A framework to estimate concentrations of potentially unknown substances by semi-quantification in liquid chromatography electrospray ionization mass spectrometry. *Anal. Chim. Acta* **975**, 30–41 (2017).
40. Raji, M. A. *et al.* Using multivariate statistical methods to model the electrospray ionization response of GXG tripeptides based on multiple physicochemical parameters. *Rapid Commun. Mass Spectrom.* **23**, 2221–2232 (2009).
41. Stavenhagen, K. *et al.* Quantitative mapping of glycoprotein micro-heterogeneity and macro-heterogeneity: an evaluation of mass spectrometry signal strengths using synthetic peptides and glycopeptides: Glycopeptide ionisation strength. *J. Mass Spectrom.* **48**, 627–639 (2013).
42. Zendong, Z., Sibat, M., Herrenknecht, C., Hess, P. & McCarron, P. Relative molar response of lipophilic marine algal toxins in liquid chromatography/electrospray ionization mass spectrometry. *Rapid Commun. Mass Spectrom.* **31**, 1453–1461 (2017).
43. Tang, W.-T., Fang, M.-F., Liu, X. & Yue, M. Simultaneous Quantitative and Qualitative Analysis of Flavonoids from Ultraviolet-B Radiation in Leaves and Roots of *Scutellaria baicalensis* Georgi Using LC-UV-ESI-Q/TOF/MS. *J. Anal. Methods Chem.* **2014**, 1–9 (2014).
44. Tu, J., Yin, Y., Xu, M., Wang, R. & Zhu, Z.-J. Absolute quantitative lipidomics reveals lipidome-wide alterations in aging brain. *Metabolomics* **14**, (2018).
45. Wu, L. *et al.* Quantitative structure–ion intensity relationship strategy to the prediction of absolute levels without authentic standards. *Anal. Chim. Acta* **794**, 67–75 (2013).
46. Yang, W.-C., Mirzaei, H., Liu, X. & Regnier, F. E. Enhancement of Amino Acid Detection and Quantification by Electrospray Ionization Mass Spectrometry. *Anal. Chem.* **78**, 4702–4708 (2006).
47. Yang, J. *et al.* A chemical profiling strategy for semi-quantitative analysis of flavonoids in Ginkgo extracts. *J. Pharm. Biomed. Anal.* **123**, 147–154 (2016).
48. Djoumbou Feunang, Y. *et al.* ClassyFire: automated chemical classification with a comprehensive, computable taxonomy. *J. Cheminformatics* **8**, (2016).
49. Ghose, A. K. & Crippen, G. M. Atomic Physicochemical Parameters for Three-Dimensional Structure-Directed Quantitative Structure-Activity Relationships I. Partition Coefficients as a Measure of Hydrophobicity. *J. Comput. Chem.* **7**, 565–577 (1986).

50. Todeschini, R. & Consonni, V. *Molecular descriptors for chemoinformatics*. (Wiley-VCH, 2009).
51. Wildman, S. A. & Crippen, G. M. Prediction of Physicochemical Parameters by Atomic Contributions. *J. Chem. Inf. Comput. Sci.* **39**, 868–873 (1999).
52. Hall, L. H. & Kier, L. B. Electrotopological State Indices for Atom Types: A Novel Combination of Electronic, Topological, and Valence State Information. *J. Chem. Inf. Comput. Sci.* **35**, 1039–1045 (1995).
53. Roy, K. & Ghosh, G. QSTR with Extended Topochemical Atom Indices. 2. Fish Toxicity of Substituted Benzenes. *J. Chem. Inf. Comput. Sci.* **44**, 559–567 (2004).
54. Liu, S., Cao, C. & Li, Z. Approach to Estimation and Prediction for Normal Boiling Point (NBP) of Alkanes Based on a Novel Molecular Distance-Edge (MDE) Vector, λ. *J. Chem. Inf. Comput. Sci.* **38**, 387–394 (1998).
55. Platts, J. A., Butina, D., Abraham, M. H. & Hersey, A. Estimation of Molecular Linear Free Energy Relation Descriptors Using a Group Contribution Approach. *J. Chem. Inf. Comput. Sci.* **39**, 835–845 (1999).
56. Pearlman, R. S. & Smith, K. M. Metric Validation and the Receptor-Relevant Subspace Concept. *J. Chem. Inf. Comput. Sci.* **39**, 28–35 (1999).
57. Kier, L. B. & Hall, L. H. *Molecular connectivity in chemistry and drug research*. (Academic Press, 1976).
58. Randic, M. On molecular identification numbers. *J. Chem. Inf. Comput. Sci.* **24**, 164–175 (1984).
59. Wiener, H. Structural Determination of Paraffin Boiling Points. *J. Am. Chem. Soc.* **69**, 17–20 (1947).
60. Wishart, D. S. *et al.* DrugBank 5.0: a major update to the DrugBank database for 2018. *Nucleic Acids Res.* **46**, D1074–D1082 (2018).
61. Wishart, D. S. *et al.* HMDB 4.0: the human metabolome database for 2018. *Nucleic Acids Res.* **46**, D608–D617 (2018).
